# Nanostructured Silica with Anchoring Units: The 2D Solid Solvent for Molecules and Metal Ions

**DOI:** 10.3390/ijms21218137

**Published:** 2020-10-30

**Authors:** Magdalena Laskowska, Oleksandr Pastukh, Andrii Fedorchuk, Mateusz Schabikowski, Paweł Kowalczyk, Marcin Zalasiński, Łukasz Laskowski

**Affiliations:** 1 Institute of Nuclear Physics Polish Academy of Sciences, PL-31342 Krakow, Poland; magdalena.laskowska@ifj.edu.pl (M.L.); oleksandr.pastukh@ifj.edu.pl (O.P.); andrii.fedorchuk@ifj.edu.pl (A.F.); mateusz.schabikowski@ifj.edu.pl (M.S.); 2 Department of Animal Nutrition, The Kielanowski Institute of Animal Physiology and Nutrition, Polish Academy of Sciences, 05-110 Jabłonna, Poland; p.kowalczyk@ifzz.pl; 3 Department of Intelligent Computer Systems, Czestochowa University of Technology, 42-200 Czestochowa, Poland; marcin.zalasinski@pcz.pl

**Keywords:** 2D solid solvent, nanomaterial engineering, silica, anchoring unit, surface functionalization

## Abstract

The ability to organize, separate and manipulate individual molecules and ions on a surface opens up almost unlimited opportunities. However, it often requires complex techniques and a proper support material. With this in mind, we show a new concept of 2D solid solvents and review a simple and efficient procedure which is based on nanostructured forms of silica with anchoring units. We describe silica supports, such as spherical nanoparticles and mesoporous silica structures, as well as review the methods for chemical modification of the surface of silica with the functional groups. Finally, we present a few particular examples of the immobilization of molecules and ions on the surface of 2D solid solvents along with the experimental investigation of the obtained materials.

## 1. Introduction

On 29 December 1959, American physicist and Nobel Laureate Richard Feynman gave a presentation entitled “There’s Plenty of Room at the Bottom” at an American Physical Society meeting at Caltech [1]. By this title, he meant a molecular level of studies, including molecular engineering. In the early 1960s, it was a melody of the future—no one could imagine how we can investigate and manipulate an individual atom or a molecule. Presently, it becomes possible since there are numerous techniques with the ability to observe even single atoms. Unfortunately, this does not mean that we have a suitable tool making for atoms and molecules tractable and easily manipulated. Of course, techniques such as a scanning tunneling microscope (STM) make it possible to manipulate atoms and small molecules; however, it is a complicated task, and it is hard to imagine using it as a part of a manufacturing process.

Indeed, an atom-by-atom assembling is complicated by conventional scientific equipment. Fortunately, it can be done by a bottom-up approach to nanotechnology [2]. According to the definition [3], it refers to the fabrication of nanoscale structures (nanoparticles) and using them as building blocks to assemble larger structures for desirable applications. We think that such a definition should be formulated in a more general way. In our opinion, the key to the bottom-up approach for molecular engineering is the design of a synthesis in such a way that atoms create assumed molecular structures through a self-organization process. This concept can be applied to manipulate the position of individual atoms and molecules while preserving the assumed distribution of the building blocks (atoms) and controlling the distance between them. Then, we can observe them as separate objects, investigate the interaction between them as a function of intermolecular distance and use the properties of individual molecules, which often differs tremendously from their bulk characteristics.

**Figure 1:**
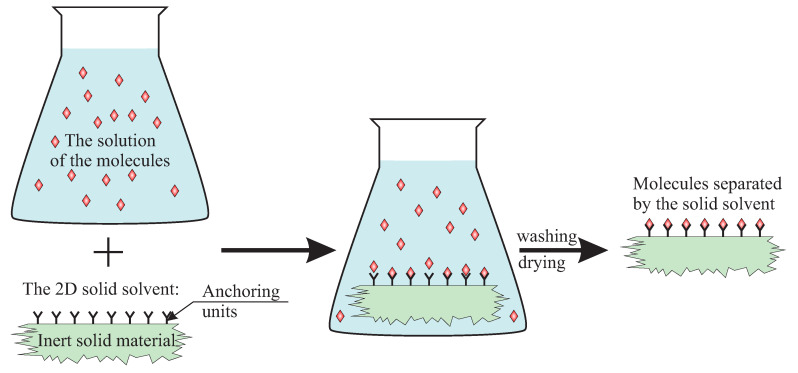
The scheme illustrating the proposed idea of a 2D solid solvent.

It sounds promising, but how to achieve this in practice? Let us imagine a solid material with regularly distributed anchoring units, separated by a specific distance, which can catch specific atoms or molecules and keep them separated. One association which comes to mind is some kind of a solvent which is able to coordinate molecules in such a way that they are separated and to create a solvation complex. In the case of a liquid, however, separated molecules are difficult to investigate since they are in a constant movement. The compound we are searching for should allow for rigid immobilization of nano-objects. This way, the objects could be used for specific purposes at specific time and place. It should be some kind of a “solid solvent” as depicted in Figure 1.

Nevertheless, such a material is not truly a solvent in the general meaning of this word since all described processes occur on its surface (on a 2D level) whereas processes in an ordinary solvent happen in its volume (or a 3D level). Thus, to understand the nature of such a material, we need to use instruments from a world of 2D (surface) physics and chemistry.

**Figure 2:**
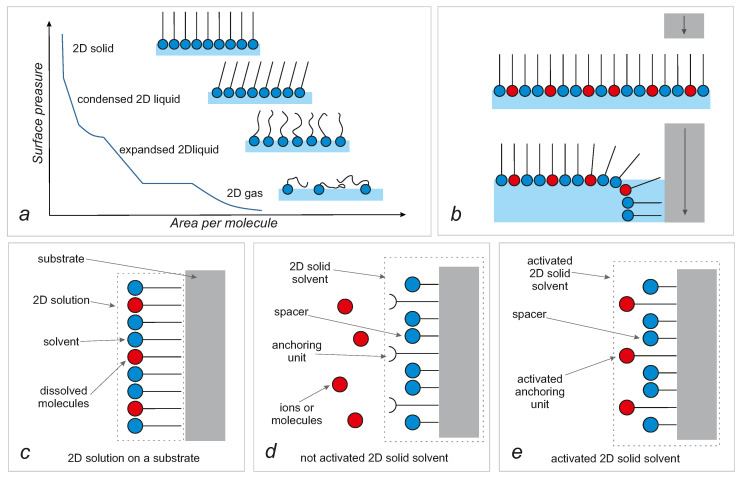
The scheme illustrating the main principles of a 2D solid solvent concept (**c**–**e**) in comparison to Langmuir monolayer (**a**) and Langmuir-Blodgett films (**b**).

Molecules on a surface may act like they are in a different state of matter—a 2D gas, liquid or solid. Such a phenomenon can be observed on the surface of surfactant solutions in which surfactant molecules are capable of the formation of a floating monolayer at the interface. This layer, under compression, can self-organize and undergo several phase changes similar to the three dimensional gaseous, liquid and solid states (Figure 2a) [4,5]. This floating monolayer (the Langmuir monolayer) can be transferred onto a solid substrate once or multiple times which results in mono- or multilayered Langmuir-Blodgett (LB) films (Figure 2b)—a 2D solid on a substrate. By using multiple surfactants, a film with a mixed composition [6] can be obtained and, thus, such a film could be treated as a 2D solution (Figure 2c).

The material we present—a 2D solid solvent (Figure 2d,e)—is similar and, from our point of view, can be treated as a 2D solution bound to a surface. Though in our case, it is much easier to obtain, more stable and is suitable for a broader range of materials. For such a material based on silica, we suggest using the following assumptions:

- a silica substrate, anchoring units and spacers are connected and must be treated as one part—a solid solvent;
- the functionalized surface of silica is treated as a deposited layer of a 2D solution;
- the surface is made only (is covered by) of anchoring units and spacers;
- spacers are treated as an analogue of a solvent and activated anchoring units—as an analogue of a solute;
- the reaction of activation of anchoring units is treated as an analogue of the occupation of sites in interstitial solid solutions but, for clarity, will be called activation.

Moreover, we emphasize that the term “solid solution” is already defined and, according to IUPAC nomenclature [7], it is a crystalline solid containing a second constituent which fits inside and is distributed in the lattice of the host crystal. Here, however, for the definition a “2D solid solvent”, we use a different term describing our approach to the problem of a solid matrix to control the distribution of molecules and the distances between them. This term can be understood as an inert solid material being able to immobilize ions or molecules on its surface and keep them separated with an assumed concentration.

Notably, it is possible to create such a material through the functionalization of a solid support material such as silica-based materials. We consider it as a starting point for the solid solvent due to the unique properties of its surface possessing numerous hydroxyl units that can be exchanged with specific anchoring units [8,9]. Moreover, the multitude of its forms makes this material extremely useful.

In this focused study, we review a few types of nanostructured silica containing specific functional units. The units can catch specific molecules and separate them from each other near the surface of the substrate. Additionally, we show an overview of possible functional units and the procedures of the functionalization of silica.

## 2. Silica as a Support

An ordinary form of silica is unremarkable to be used as a support for anchoring units. This is mainly because of the large size of individual grains which provide low specific surface area [10]. In its original form, even with anchoring units, silica could not immobilize a significant number of molecules or atoms. Fortunately, there are numerous nanostructured forms of a silicon dioxide with a more developed surface.

### 2.1. Spherical Nano-Silica

**Figure 3:**
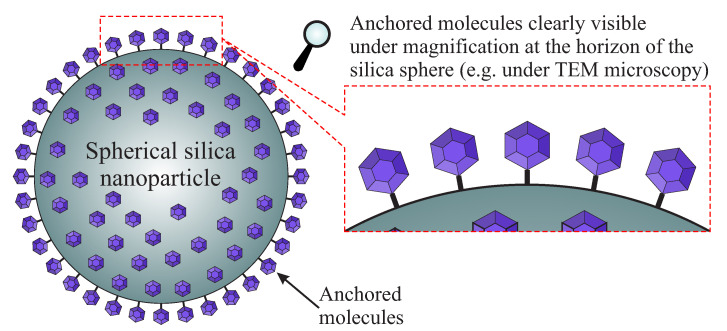
The concept of direct observation of separated molecules at the horizon of spherical silica support.

Ordinary silica became revolutionary for the first time in the Sixties when Werner Stöber introduced the procedure for the fabrication of nanostructured SiO${}_{2}$ [11]. To this day, this wet-chemistry route of the preparation of silica nanoparticles of a controllable and uniform size remains the most common synthetic approach. Thanks to this method, it became possible to fabricate silica particles with diameters ranging from 30 to 2000 nm depending on conditions and the proportions of reactants [11,12,13,14]. The resulting spherical silica nanoparticles possess a significantly larger specific surface area than ordinary bulky silica but not as large as the porous one which we will discuss in a different section. One can ask why such a form of SiO${}_{2}$ is worth consideration as a solid solvent. A solid spherical form assures that all functional units will be placed on the external surface of the material. This is important mainly for the separation of larger molecules (with a diameter larger than 1 nm). In such a case, the distribution of attached molecules can be directly observed using transmission electron microscopy (TEM). To roughly estimate distances between the separated molecules, one can observe the horizon of the spheres as presented in Figure 3. This method is not very accurate but is direct and can confirm the presence of separated molecules unambiguously.

**Figure 4:**
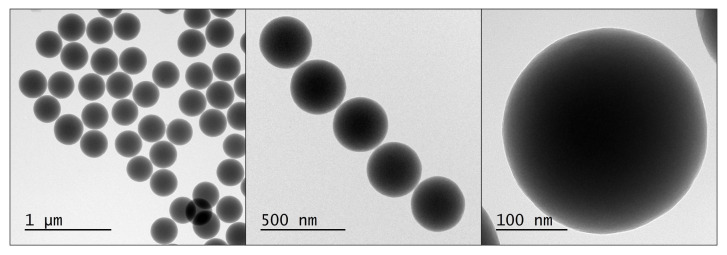
TEM micrographs of spherical silica prepared according to the Stöber protocol.

The first attempt to obtain nanosilica with a uniform size distribution was made in 1956 by Kolbe. As he described in his thesis [15], uniform spherical silica can be obtained in a reaction of tetraethyl orthosilicate (TEOS) in an alcohol solution with water in the presence of certain bases. The author emphasises the importance of high purity of the reagents for a successful synthesis. Unfortunately, the presented reaction was not repeatable and did not lead to the formation of uniform nanosilica. It was, however, a starting point for Stöber and his colleagues to develop an efficient method for fabrication of spherical nanosilica. In the reaction presented in the original article [11], ammonia was used as the catalyst promoting the formation of spherical particles. Silica was created as a result of hydrolysis of alkyl silicates and subsequent condensation of silicic acid in alcoholic solutions. The authors used tetramethyl orthosilicate (TMOS) and tetrapentyl orthosilicate (TPOS) as precursors of the silica and various combinations of water and alcohols (propanol, n-butanol, ethanol and methanol). Depending on the composition of the reactants, various nanoparticles were obtained with different size distributions. The reaction was the fastest when methanol was used, whereas the slowest was in the presence of n-butanol. The obtained particle size under comparable conditions was the smallest in methanol and the largest in n-butanol. When the higher alcohols were used, the size distribution of the particles was wider than for ethanol and methanol. As for the silica precursors, TMOS reaction was faster and resulted in smaller silica spheres than TPOS. The systematic investigation of the influence of the initial sol composition on the resulting nanoparticles can be found in their original article [11]. An optimized Stöber protocol leads to the fabrication of uniform 300-nm silica spheres, as presented in Figure 4. The detailed procedure is shown in Supplementary Information.

The work of Stöber was extended by Bogush et al. [16] who optimized concentration of the reagents which resulted in the formation of monodisperse silica particles. The authors applied various proportions between ethanol, ammonia, water, and TEOS. Moreover, various temperature conditions were tested regarding the properties of the resulting nanoparticles. The obtained size of the nanoparticles was tuned from 50 to 800 nm. The smallest standard deviation of the sizes was achieved for the average size greater than approximately 300 nm. For the maximum achievable size, a broad or multimodal particle size distribution was often obtained. The temperature effect was studied from 9 to 55 °C. Outputs indicated that the particle size decreases monotonically as temperature increases. Interestingly, the stirring rate did not influence the resulting material.

For the set temperature of 25 °C and concentration ranges of 0.1–0.5 M TEOS, 0.5–17.0 M H${}_{2}$O and 0.5–3.0 M NH${}_{3}$, the authors established an equation for the diameter of the obtained silica spheres (d—in nanometers) in the following form:

(1) $$d=A\left[H_{2}O\right]\cdot 2\exp{\left(-B[H_{2}O]\right)}^{2}$$

In Equation (1), the constants are:

(2) $$A={\left[TEOS\right]}^{\frac{1}{2}}\left(82-151\left[NH_{3}\right]+1200{\left[NH_{3}\right]}^{2}-366{\left[NH_{3}\right]}^{3}\right)$$

and

(3) $$B=1.05+0.523\left[NH_{3}\right]-0.128{\left[NH_{3}\right]}^{2}$$

The reagent concentrations are in mol/L. In the same article, the authors presented the impact of reagents on the synthesis [16]. With increasing particle size, however, the distribution of the particles size was bimodal.

There were attempts to obtain larger silica particles (>800 nm) with homogenous distribution of their sizes [17]. It was done by preventing the formation of secondary particles during the sol precipitation. This was achieved by a slow rate of adding the reactant and a proper mixing rate. Interestingly, the addition of higher volumes of TEOS (than those assumed by Stöber) also resulted in large particles with a uniform size [14].

**Figure 5:**
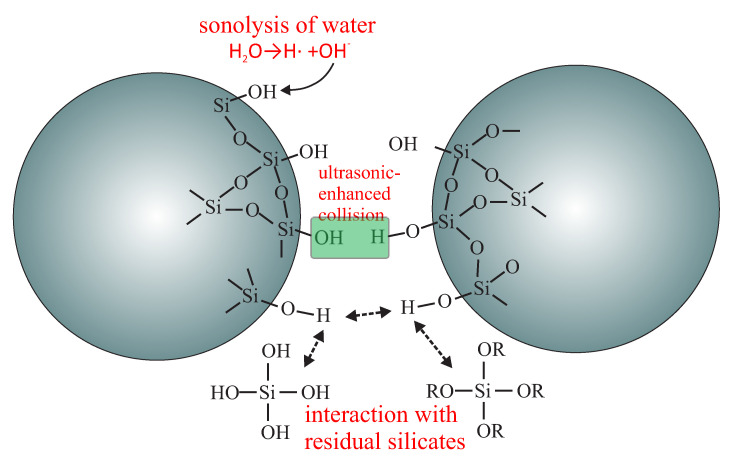
Proposed mechanism for agglomeration of silica spheres through the ultrasonic-enhanced collisions.

The influence of the conditions, such as the level of ultrasonic stirring, on the resulting spherical silica, were tested in [18]. By using an ultrasonic homogenizer during the reaction, the particles size is influenced as follows: the particles grow larger with ultrasounds than when mechanical stirring is used. Moreover, ultrasonic stirring induces the agglomeration of particles. This is an interesting effect and was investigated by the authors on the basis of ultrasonic-enhanced collisions among spheres, as shown in Figure 5.

#### The Nanostructure and the Mechanism of the Formation of Stöber Silica Particles

The fabrication of monodisperse colloidal silica spheres follows the reactions:

(4) $$Si{\left(OC_{2}H_{5}\right)}_{4}+H_{2}O\overset{NH_{3}}{\underset{alcohol}{\to }}Si{\left(OH\right)}_{4}+4C_{2}H_{5}OH$$

(5) $$Si{\left(OH\right)}_{4}\overset{NH_{3}}{\underset{alcohol}{\to }}SiO_{2}↓+2H_{2}O$$

However, the chemical steps presented above are general and can be treated as an oversimplification. The real mechanism of the formation of silica spheres is more complex. To this day, there is no agreement concerning the mechanism of the formation of particles in the Stöber solution. The first attempt to the clarification of this mechanism and development of mechanistic models of particle growth was made in 1988, and two extreme models were proposed: monomer addition and the controlled aggregation model.

The monomer addition model was proposed before the controlled aggregation model by Matsoukas and coworkers [19,20]. Based on experimental results, the authors formulated a few important conclusions:

- the rate of hydrolysis controls the silica growth process even for high water-to-orthosilicate ratios;
- reaction rates are faster in methanol than in ethanol;
- particles grow larger in ethanol than in methanol under the same conditions;
- ammonia increases the reaction rate and promotes the formation of larger particles;
- low water concentration favors the production of larger particles, but the excess of water has the opposite effect.

The authors concluded, that the growth is characterized by an incubation period during which the nucleation takes place. After this period, no significant nucleation occurs [19]. Further growth of silica depends on the addition of hydrolyzed monomers to the surface of oligomers.

The creation of kernels and the growth of silica spheres were described in details in [20]. Such a model was claimed as a correct and fitted well to the experimental results presented in [14].

In the controlled aggregation model [16], the authors, based on experimental results, presented three basic findings leading to the postulated mechanism:

- the growth mechanism is self-sharpening—small particles grow faster than large particles;
- the growth mechanism does not alter dramatically during the reaction—the density of the silica particles and their external morphology are independent of the particle size;
- the growing particles must, at some point, achieve colloidal stability to obtain monodisperse particles.

On this basis, the authors postulated that particle growth occurs primarily through an aggregation mechanism. Next, the monodispersity occurs due to size-dependent aggregation rates (the probability of aggregation between two particles of the same size decreases while the particles grow). According to Deryagin-Landau-Verwey-Overbeek theory [21], the repulsive interparticle potential has its origin in charges bound to particle surfaces. On this basis, one can conclude that silica nuclei formed in a supersaturated solution are colloidally unstable and thus aggregate. Since the growth mechanism is self-sharpening, the distribution of the particle size shifts towards larger particles. At the same time, larger particles do not agglomerate with each other and with smaller particles. A quasi mono-sized dispersion is achieved. This mechanism was further developed in another article by Bogush et al. [22,23].

Preliminary tests of the structure of the particles were performed in 1988 [16]. The density measurements of the material indicated micropores inside the particles. The calculated porosity of the material was 11–15%. This finding was significant for the explanation of the mechanism of the formation of spherical silica and its nanostructure. The porosity can be decreased by heating at 800 °C for 3 h or longer in air [24].

The morphology of the particles was tested by means of ${}^{13}$C and ${}^{29}$Si nuclear magnetic resonance spectroscopy (NMR) [25]. The main finding was that the siloxane structure of the silica particles was composed of approximately 60% Q${}^{4}$, 35% Q${}^{3}$, several percent Q${}^{2}$, and that several percent of the ethoxy groups never leave the TEOS molecules (the notation of silanol groups is explained in Section 2.2.3). This indicates that the synthesis is not as simple as presented in Equations (4) and (5). The same authors in their different article [26] deliberated on the growth mechanism of silica spheres with a bimodal size distribution. The main conclusion was that particle formation depends on the aggregation process of tetraethyl orthosilicate. This process is strongly influenced by the surface potential of the silica particles and by the ionic strength of the reaction medium.

**Figure 6:**
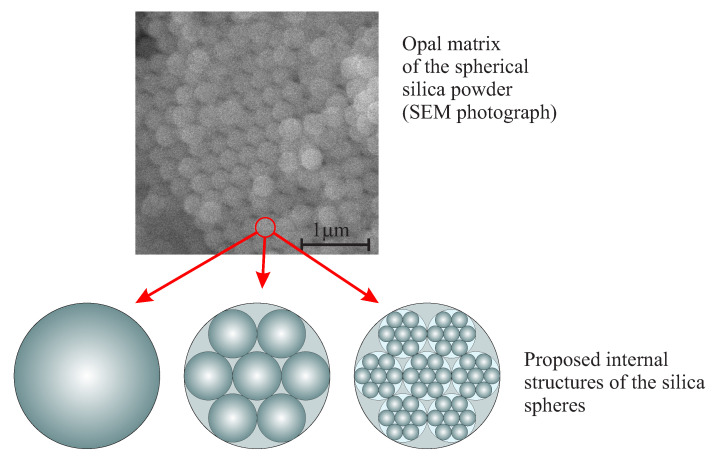
Possible simple models of the interior structure (opal matrix) of silicon dioxide spherical particles.

**Figure 7:**
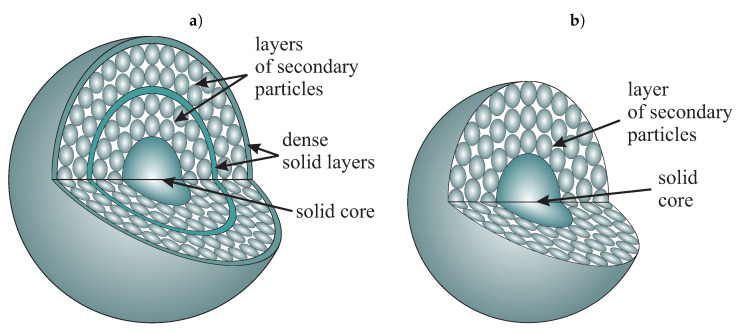
A schematic representation of the multi-layer structure of silicon dioxide particles: a two-layer model of the large particle (**a**), and a shell-like model of the silica grown by the multistage SFB method (**b**).

Further development of the mechanism of silica formation was presented in [27,28] along with a detailed analysis of the structure of spherical silica. As a starting point, the authors consider the opal structure of, both the powder and its internal architecture (model) [29] as it is shown in Figure 6. It is maintained, that the porosity derives exclusively from octahedral voids. The porosity measured for the silica dioxide spheres presented in the literature for various diameters [16,30] do not point at any particular model. The authors concluded that silica particles could have different structures and density depending on their size. Moreover, the authors established that large particles can contain a central core that is composed of primary particles surrounded by several layers of secondary particles smaller than the core (Figure 7a [28]).

Silica particles, obtained during the multistage Stöber–Fink–Bohn (SFB) method [27], are characterized by a more complex structure composed of a solid silica core and a few layers of secondary particles (<100 nm) which are separated by solid shells (shown in Figure 7b).

However, scientists did not agree upon neither the exact structure of the Stöber silica nor the formation mechanism. The presented models still need to be verified experimentally, and it is a vital task of modern science.

### 2.2. Mesoporous Silica Structures

#### 2.2.1. A Brief Review of Porous Silica Types and Structures

Porous silica-based materials belong to a large group of inorganic materials which have an open-pore structure and are characterized by a sizeable surface area. Based on the IUPAC classification [31], porous silicates are classified based on a pore diameter *d* and can be divided into microporous (d<2 nm), mesoporous (2<d<50 nm) and macroporous (d>50 nm) materials. Zeolites are well-known representatives of the class of microporous materials [32] which provide excellent catalytic properties due to their crystalline aluminosilicate network. However, the dimensions of pores in zeolites and their accessibility are limited to the sub-nanometric scale (<1.3 nm). Due to the small pores, their application is limited only to the small molecules. Macroporous materials, such as traditional ceramics and cement, mostly have an unordered structure—channels or pores are irregular and have no long-range ordering. Therefore, the most promising material, with controlled pore size and their arrangement seems to be mesoporous silica. The presence of mesopores provides selectivity in size and shape for guest molecules enhancing host-guest interactions and, thus, can be applied in numerous fields.

Mesoporous materials originate from the first research in 1969 by the group of American scientists from Sylvania Electric Products Inc. in which the procedure for the preparation of low-density silica was described [33]. However, the main goal of the original patent was only to show the formation of low bulk density silica and not its porosity. Only a few of the remarkable properties of the material were discovered [34]. In the late 1980s and early 1990s, independent studies on the synthesis of mesoporous silica materials were performed by a Waseda University group (Japan) [35]. The authors obtained a three-dimensional silica network with a uniform pore-size distribution. Their synthesis was based on a layered silicate, kanemite (NaHSi${}_{2}$O${}_{5}$·3H${}_{2}$O) and using a quaternary ammonium surfactant as a template. Based on this work, S. Inagaki succeeded in the synthesis of highly ordered mesoporous silica with hexagonal symmetry (also derived from the kanemite) named FSM-16 (Folded Sheets Mesoporous materials) [36,37]. By using templating with surfactant aggregation and precise pH control, the authors could produce materials with a tailored mesoporous structure.

**Figure 8:**
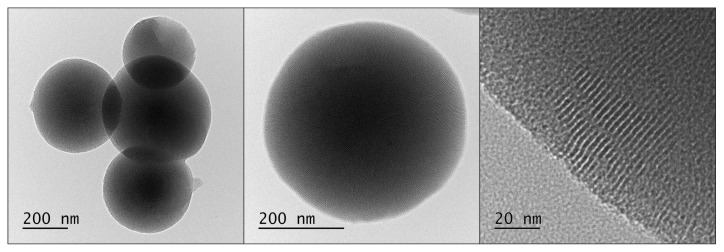
TEM micrographs of MCM-41 silica.

Around the same time, parallel to the Japanese group, researchers in Mobil Corporation independently synthesized mesoporous materials with hexagonal (p6mm), cubic (Ia3d) and lamellar (p2) symmetry [38,39]. The family of mesoporous molecular sieves was named M41S. It is believed that the report of M41S materials and especially the discovery of the MCM-41 (Mobil Composition of Matter No.41) gave rise to an active research and aroused wide interest in mesoporous structures. The most studied MCM-41 silica exhibits a two-dimensional hexagonal array of uniform channels with the diameter, which can be controlled, between 1.5 and 10 nm. In contrast to FSM-16 materials, for which a “folded sheet” formation mechanism from a layered silicate is proposed, the Mobil group developed a “liquid crystal templating mechanism” (LCT). Instead of traditionally used in zeolite synthesis single-molecular or cationic templates, organized assemblies of molecules were first used as templates which made it possible to create a material with favorable characteristics: the pore walls of about 0.8 nm in thicknesses, the specific surface area between 1000 m${}^{2}$/g to 1200 m${}^{2}$/g and pore volume of approximately 1 cm${}^{3}$/g. The MCM-41 material could be synthesized in the form of spherical particles, as it was shown in Figure 8.

**Figure 9:**
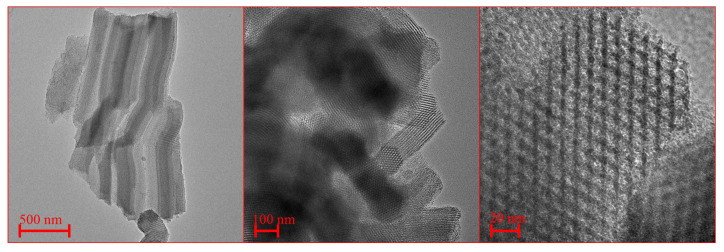
TEM micrographs of SBA-15 silica.

However, the mechanical properties of MCM-41 were not satisfactory [40]. Due to the thin walls, the specimens were not durable, and the structure was prone to damage [41]. Moreover, after the removal of a surfactant, the structure could deform in time. For this reason, virtually since the discovery of M41S, many scientific groups are involved in the investigations of new synthesis pathways which led to more stable and more complex nanostructures with a wider compositional range. Various other types of mesoporous silica nanomaterials have been developed. P. T. Tanev et al. proposed the synthesis of HMS (Hexagonal Mesoporous Silica) using neutral amines as templates [42]. S. A. Bagshaw et al. synthesized the disorganized mesoporous materials MSU-1 (Michigan State University) using polyethylene oxide (PEO) [43]. However, the most exciting novel development, after the discovery of the initial pathways, was made by the Santa Barbara group at the University of California [44]. The hexagonal well-ordered mesoporous silica SBA-15 (Santa Barbara Amorphous) significantly expanded the application of mesoporous materials. Before this discovery, the pore size of an ordered mesoporous silicon dioxide was limited to approximately 10 nm with the wall thickness of about 1 nm. The use of Pluronics type triblock copolymers, such as P123 (poly(ethylene glycol)${}_{20}$-poly(propylene glycol)${}_{70}$-poly(ethylene glycol)${}_{20}$) as a surfactant, noticeably expanded the available range of parameters of the obtained mesoporous materials [45]. This method made significantly larger pore size, from 4 nm to about 40 nm, the wall thickness of up to 6 nm, and the specific surface area of 800–1000 m${}^{2}$/g possible. The structure of SBA-15 silica is shown in Figure 9. Moreover, the resulting material, the SBA-15 silica, is characterized by high mechanical, thermal (>900 °C) and hydrothermal stability [41,46]. Since this initial publication, numerous researchers used SBA-15 instead of MCM-41 in their investigations.

#### 2.2.2. Formation Mechanism and Structure

The LCT mechanism, proposed by Mobil Corporation scientists, is a typical synthesis for the formation of mesostructure materials which initially included two basic approaches, such as True Liquid Crystal Templating [47] and Cooperative Self-Assembly mechanism [48,49], but also could have several variations. In general, the necessary components for such structures are inorganic precursors (as a source of a silica framework), organic template molecules (a surfactant) and a solvent with an acid or basic catalyst. An important factor that plays a crucial role in forming of the final structure, pore sizes and surface areas of mesoporous material is a surfactant. The formation of an organic-inorganic liquid-crystal mesophase is the result of surfactant molecules and inorganic species self-assembly that polymerize (or condense) under certain conditions.

**Figure 10:**
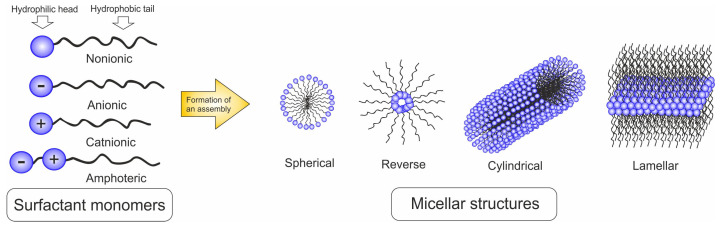
Types of surfactants and micellar structures.

The characteristic molecular structure of surfactants consists of a structural group that has very little attraction for the solvent (the lyophobic group) and a group that has a strong attraction for the solvent (the lyophilic group). Such structure is known as an amphipathic [50]. Surfactants are classified depending on the nature of the hydrophilic group. They may be anionic (the surface-active portion of the molecule possesses a negative charge), cationic (the surface-active portion bears a positive charge), amphoteric (both positive and negative charges may be present in the surface-active part) and nonionic (the surface-active portion bears no apparent ionic charge) (Figure 10).

The fundamental property of a surfactant lies on the formation of a micelle—an organized auto-assembly formed in a liquid and composed of amphiphilic macromolecules [51]. The formation of the micelles, as well as the aggregation into the corresponding structure, depends on the concentration of a surfactant. It is characterized by a critical micelle concentration (CMC) [52]. At a low micelle concentration (below CMC), molecule ordering is not possible. Spontaneous assembly of micelles can happen at slightly higher (above CMC) concentrations. When a sufficient number of micelles in the solution is reached, they start to build a structure in several ordered geometric arrangements depending on the shape of the individual micelles [53]. Because of, both the ordered molecular arrangement of solid crystals and the mobility of liquids, these packing arrangements were called liquid crystals. The formation of micelles, their shape and the aggregation into liquid crystals depend on the concentration of a surfactant [54]. Spherical micelles arrange into cubic liquid crystals, cylindrical micelles into hexagonal liquid crystals and lamellar micelles form lamellar liquid crystals (Figure 10). Spherical geometry is the most favorable arrangement because of the most efficient minimalization of surface energy.

The first ordered mesoporous materials, such as MCM-41 and FSM-16, were prepared from cationic surfactants, such as quaternary alkylammonium ions, under alkaline conditions. The formation of the inorganic-organic hybrid mesophase is based on electrostatic interactions between the positively charged surfactant molecules and the negatively charged silicate species in solutions. The primary synthetic route involves the direct co-condensation of anionic inorganic species ($I^{-}$) with a cationic surfactant ($S^{+}$) and is abbreviated as $S^{+}I^{-}$ interaction [49,55] (Table 1). The most widely used surfactant is CTAB (cetyltrimethylammonium bromide). Optimal CTAB concentration is needed for the formation of a liquid-crystal mesophase. In the presence of silica anions, the micellar solution of CTAB transforms into a hexagonal lyotropic phase (MCM-41) or cubic phase (MCM-48) [56]. The pH of the synthesis media can be from very basic (the optimum pH value is in the range of 9.5–11.5) to near neutral. The silicon resource may be either organic silicon compounds, such as TEOS, TMOS or tetrabutyl orthosilicate (TBOS), or inorganic compounds such as amorphous silica, soluble silicate.

The morphology SBA-15 depends directly on the conditions of a synthesis. The typical synthesis of SBA-15 is performed by using nonionic surfactants (typically containing EO block) as templates. One of the widely used templates is the family of poly(ethylene oxide)–poly(propylene oxide)–poly(ethylene oxide) (PEO–PPO–PEO) triblock-copolymers. One of them is Pluronic P123, EO${}_{20}$PO${}_{70}$EO${}_{20}$ (with the average molecular weight of 5800). Other surfactants (P104, P85, P65, L65, etc.) are also applicable, but they require a higher reaction temperature. P123 is an amphiphilic molecule where the PEO parts are hydrophilic, and PPO part is hydrophobic. The amphiphilic character of P123 results in the formation of spherical micelles in water with EO chains towards the water and PO chains in the direction of the core of micelles.

In contrast to the cationic surfactants used in the preparation of MCM-41, the nonionic surfactants have a higher concentration in the synthesis media. In general, the use of triblock copolymers expands the accessible range of pore sizes. Mesoporous silica obtained with the use of triblock copolymers possesses large uniform pores and thick walls, the latter providing high thermal stability and improved hydrothermal stability compared with mesoporous silica prepared by using low-molecular-weight surfactants. In a basic environment, it is difficult to prepare SBA-15 with nonionic surfactants. For this, a strongly acidic medium, such as 2.0 M of HCl solution, and the optimum pH value lower than 1.0 value is required. At such conditions, protonated PEO chains of surfactants interact with cationic silica species by weak electrostatic interactions mediated by the negatively charged chloride ions. This pathway is termed $S^{0}H^{+}X^{-}I^{+}$ [57,58] (Table 1). Here, the surfactant-silica interaction is mediated by the counterion $X^{-}$ neutralizing the repulsion between positively charged surfaces through hydrogen bonding. TEOS or TMOS, in combination with a diluted acidic aqueous solution of Pluronic-type triblock copolymer, are used as silica precursors. In comparison to MCM-41, SBA-15 has a more complex framework structure [59]. In the preparation of SBA-15, the EO segments of the PEO-PPO-PEO template can enter the silica walls and form hybrid inorganic–organic composite frameworks. Upon removing the template by calcination, micropores and small mesopores, of the size of about 1–3 nm, are generated in the framework connecting the main pore channels. That is why the framework structure of SBA-15 and MCM-41 is quite different.

#### 2.2.3. The Surface of Silica

An active silica surface characterized by a large specific area is of great importance for numerous applications. However, since different silica compositions and structures are very diverse, the surface and framework properties are also extremely varied.

**Figure 11:**
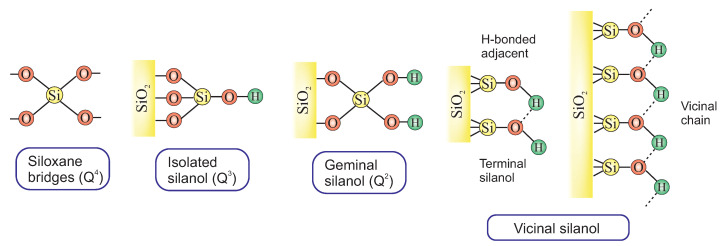
Various types of the surface of silica.

When viewed on the inside of the silica framework (bulk), the Si–O–Si bonds are dominant when four other silicon atoms surround one silicon atom. However, a completely different situation occurs on a surface of silica where framework coordination may be partly changed to Si–OH bonds. The classification of the diverse silica surface could be done according to the number of silanol and siloxane functionalities [54]. Silicon atoms are referred to as $Q^{n}$ groups (1 ≤n≤ 4), where n defines the number of neighboring silicon atoms (Figure 11). Thus, a $Q^{4}$ group corresponds to a structure like TEOS where all four oxygen atoms are bound to another silicon atom; in $Q^{3}$ group, a silicon atom is linked to three other silicon atoms through the oxygen and have one “single” silanol. In the $Q^{2}$ group, a silicon atom is oxygen-linked only to two other silicon atoms, and two isolated silanols are present. There is also the possibility of the formation of vicinal or bridged silanols where two isolated silanol groups attached to two different silicon atoms are bridged by H-bond [60].

The understanding of the surface structure of nanosized silica is the critical point for the synthesis of functionalized materials since silanol groups may act as functional groups in chemical reactions or can be used to attach molecules having other functional groups. Moreover, the knowledge of the exact number of silanols on the silica surface is essential in areas such as catalysis [61,62] and adsorption [63,64]. The nature and distribution of silanol groups on silica surfaces are highly sensitive to the synthetic route and strongly dependent on the temperature of any post-synthesis treatment [65].

There are different methods which can be used to characterize and quantify the density of silanol groups of both amorphous and mesostructured silica materials. Among others, the methods include thermogravimetric analyses, diffuse reflectance Fourier transform infrared spectroscopy (DRIFT), ${}^{29}$Si and ${}^{1}$H NMR, microcalorimetry and their combinations [66,67,68,69,70,71]. For example, the first study of the surface of mesoporous MCM-41 [72,73] and SBA-15 [44] identified the presence of different types of silanol groups in template-free samples: isolated silanols, geminal silanols and interacting vicinal pairs. However, there is a possibility for the formation of other vicinal chains, such as H-bonded adjacent silanols, terminal silanols in H-bonded chain or vicinal chains containing more than one pair of mutually H-bonded OH groups (Figure 11). Some of them, however, are less reactive due to a strong interaction with neighbouring silanols. By comparing MCM-41 with SBA-15, it was found that the latter possess a larger amount of surface silanol groups due to the microporous framework structure [74]. However, more than half of these groups are located in the micropores in the pore walls and are not available either for surface modification or small silane. Cubical pore systems, like SBA-16 and MCM-48, systematically have a larger amount of silanol groups on the surface in comparison to MCM-41 and SBA-15. This is explained by the fact that a cubical pore system is accessible from three dimensions while a hexagonal pore system just from one.

## 3. Chemical Modification of Silica Surface

In order to transform the nanostructured silica into a 2D solid solvent, its surface needs to be modified to selectively adsorb desirable ions or molecules. In other words, we need to convert the surface hydroxyl units (see: Section 2.2.3) into the appropriate anchoring groups. Since a larger surface leads to the possibility of placing more anchoring units with a homogenous distribution, the role of nanostructurization is essential. Moreover, apart from the external surface of nanoparticles, also the inner structure can be functionalized, which will increase the available locations to attach ions and molecules.

In this section, we present the methods for the modification of silica surface with organic units which could be used to obtain the 2D solid solvents. In the literature, all methods in which organic functional groups can be attached to the surface of silica are divided into two main groups:

- through a reaction between pristine/modified silica surface functions and organosilanes or organic molecules (grafting);
- incorporation of functional groups via sol–gel material synthesis methodology (co-condensation).

Moreover, the already attached organic groups can also be modified, or even grafting can be performed to them [75]. In this section, we present a short review of both primary connection of organic groups to silica surface and their post-attachment modification in order to obtain highly reactive units which enable the immobilization of ions and molecules.

### 3.1. Grafting

Grafting is a broad term for post-synthesis modification techniques where, in contradiction to co-condensation, the material for a further functionalization is synthesized during a separate step. Grafting of silica is based on the modification of material surface through the reaction of free –Si–OH (silanol) groups which are present on the surface with a typical density of approximately 2–3 Si–OH groups per nm${}^{{}_{2}}$ [76]. There are two main ways in which grafting of a silica surface can be done, namely:

- direct grafting of silanol groups of silica—in this approach, organic or silica-organic reagents are attached to the surface via a condensation process;
- grafting of non-silanol groups—first, silanol groups of silica are modified with other chemical groups, such as Si-Hal or Si-H, and afterwards, a standard grafting is performed with desired molecules.

Grafting became widely used due to (but not limited to) the following advantages:

- a large variety of functional groups can be introduced to a material;
- for mesostructured silica, the original structure of the silica phase is usually retained—one can use pre-prepared samples with well-known structures (in the case of co-condensation, the introduction of some trialkoxysilans in the existing preparation technique could affect the formation of the assumed mesostructure);
- there is no strict requirement in the synthesis of complex silica matrix—numerous silica matrices can be purchased from commercial sources.

However, grafting has some disadvantages as well. In the case of porous structures, the obtained materials can have their porosity decreased after the process. Moreover, since on the initial stages of the reaction, grafting reagents react preferentially at the external surface of silica particles (near the pore openings), the diffusion of farther molecules into the pores can be restricted. As a result, it can lead to a non-homogeneous distribution of organic groups in the pores or, in extreme cases of large functional groups and small pores, pore blocking.

#### 3.1.1. Grafting of the Silanol Groups

**Figure 12:**
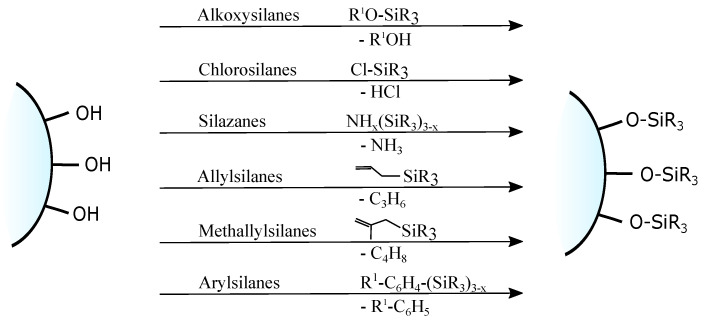
Grafting of silanol groups with various organosilanes.

Usually grafting of silica surface is done with the use organosilanes (RSiX${}_{3}$, R${}_{2}$SiX${}_{2}$, or R${}_{3}$SiX, where R is a desirable organic group and X is a leaving group). In traditional techniques for surface modification, alkoxy- or chloro- groups are used as leaving groups. The examples of the possible organosilanes usage are depicted in Figure 12.

Commercially available silanes, which can be used for grafting of the silica, are based mostly on trichlorosilanes (Cl${}_{3}$Si-R), trimethoxysilanes ((CH${}_{3}$O)${}_{3}$Si-R), or triethoxysilanes ((C${}_{2}$H${}_{5}$O)${}_{3}$Si-R). From the reagents above, trichlorosilanes are more reactive than the trimethoxy- and triethoxysilanes [77]. Another standard reagent is trimethylsilyl chloride—TMSCl ((CH${}_{3}$)${}_{3}$SiCl) which is used for silanization of silica and glass surfaces including the silanization of laboratory glassware to make it more hydrophobic. Due to its common usage in organic chemistry to protect some of the functional groups, TMSCl is one of the most accessible reagents for the functionalization of the surface of silica.

Nevertheless, chlorosilanes and alkoxysilanes are most commonly used, there are also numerous different reagents, which were investigated for modification of silica surface, namely mono- di- and trisilazanes [78], allylsilanes [79], methallylsilanes [80], arylsilanes [81] and other [82]. The number of leaving X groups in the reagent is typically equal to one or three with the most common composition of R(CH${}_{3}$)${}_{2}$Si-X or R-SiX${}_{3}$.

Monofunctional organosilanes have only one leaving group in the molecule and, as a result, only one type of bonding is possible. This results in better reproducibility of the surface grafting. On the other hand, when using these reagents, the overall surface coverage is lower due to the sterical hindrance from three functional R groups absent in the case of trifunctional organosilanes. In the last ones, possessing only one R group, all three leaving groups can react both with surface silanol groups and other organosilanes. This can be beneficial due to the formation of intra-monolayer cross-linking. It can also be detrimental because of the possibility of homocondensation (oligomerization) in a solution [83].

The main advantage of using silane precursors is their commercial availability and strong connections with the surface of silica they could make in addition to the great variety of the functional moieties which can be attached to the silane “core” [77].

Nevertheless, using silane-based reagents involves several practical problems [84], since these reagents are:

- susceptible to competing side reactions,
- responsive to moisture,
- tend to undergo intermolecular polymerization/polycondensation,
- can form multilayers.

**Figure 13:**
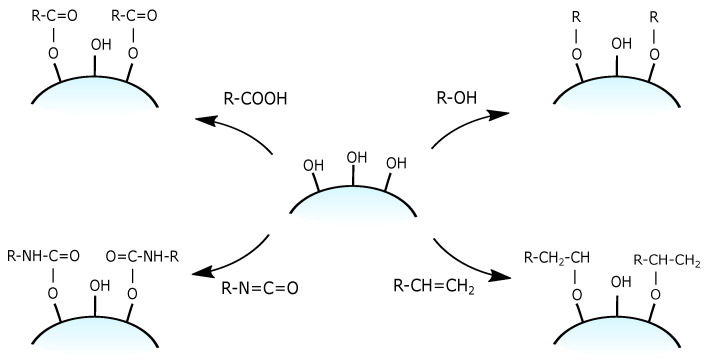
Grafting of silanol groups with simple organic compounds.

As an alternative to the silane-based reagents to modify the surface of silica, various simple organic molecules were proposed [83] such as alcohols [85], alkenes [86], isocyanates [87] and carboxylates [88] with the possible formation of corresponding Si-O-C or Si-O-CO- bonds (see: Figure 13). Nevertheless, due to the possibility of hydrolysis in the presence of water, they can only be used in relatively dry environments which significantly limits their usage.

#### 3.1.2. Grafting of Chlorinated Silica

**Figure 14:**
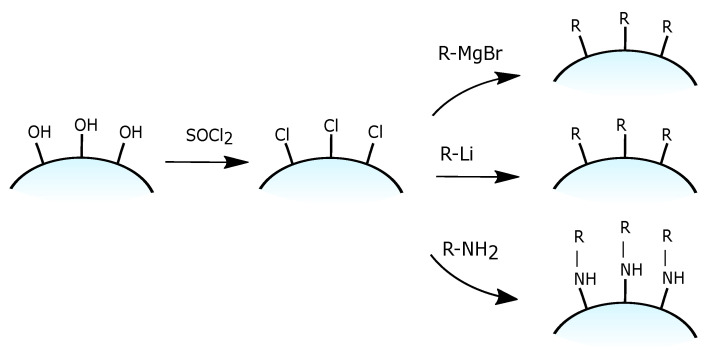
Grafting of chlorinated silica.

Condensation via chlorination is another type of post-synthesis modification of silica. Unlike ordinary grafting, in this method, the modification of silica surface consists of two stages, as presented in Figure 14. The first stage is a chemical activation through the formation of active Si-Cl (Si-Hal) bonds via the reaction with thionyl chloride–SOCl${}_{2}$ [89] or another halogenation agent such as CCl${}_{4}$ [90] and (COCl)${}_{2}$/POPh${}_{3}$ in CHCl${}_{3}$ [91]. During the second stage, grafting of previously obtained Si-Cl groups with corresponding organics is performed. Since halogenation agents are extremely reactive with water, this reaction must be carried out in an anhydrous environment. From the practical point of view, it means that before the reaction, silica must be dried (up to 350 °C) to remove the adsorbed water. The obtained activated material must be kept in an enclosure with no access to humidity.

In the next stage, it can be modified in a dry diethyl ether using desired Grignard reagent or other organometallic reagents, obtaining stable Si-C bonding [92,93]. Another possible reaction is the modification with amines via Si-N bond formation [94]. Their good availability and low cost (in comparison with trialkoxysilane-based reagents) are the main advantages of this approach. On the other hand, this method also has a wide list of drawbacks, such as the necessity of hazardous reagent usage as well as the formation of dangerous side-products (HCl, SO${}_{3}$, Cl${}_{3}$CO) and a limited range of functional groups which can be introduced [95,96].

#### 3.1.3. Grafting of a Silica Hydride

Silica-hydride materials, which are also known as Type C silica—are based on ordinary silica with one fundamental difference—their surface is covered with less polar silicon-hydride (Si–H) groups which replace 95% of the polar silanol Si–OH groups on their surface. Type C silica is known as promising chromatographic support materials [97] as well as an intermediate in the synthesis of organically modified silica. This modification can be done via the hydrosilation process producing a bound stationary phase in which desirable organic moiety is bound to the surface of silica by a stable Si–C bond.

**Figure 15:**
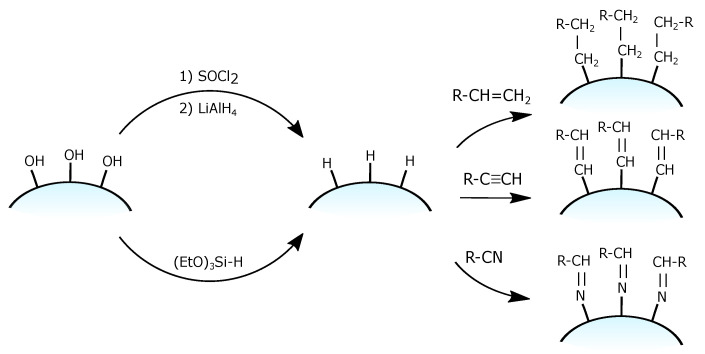
Grafting of a silica hydride.

Generally, the attachment of organic moieties via this method is a two-step process [98], as depicted in Figure 15. During the first step, a monolayer of a hydride is obtained on the surface. Unlike the chlorinated silica, the hydride silica, due to its greater stability, can be stored for long periods without noticeable decomposition. On this stage, there are two main synthetic approaches to obtain a silica hydride monolayer [99]:

- the reduction of chlorinated silica Si-Cl groups to Si-H using lithium aluminium hydride LiAlH${}_{4}$ [100] in completely dry conditions to avoid Si–Cl bond hydrolysys;
- the single-step grafting of hydride monolayer on ordinary silica using the condensation of silane reagents (most commonly with triethoxysilane (C${}_{2}$H${}_{5}$O)${}_{3}$Si-H) [101].

The second step consists of the catalyzed attachment of a desired organic group to the silica-hydride surface via a hydrosilation reaction. The most common groups for this reaction are terminal olefins. Nevertheless, there are also works on the attachment of different groups with multiple bonds such as nonterminal alkenes, alkynes [102], nitriles [103] (with the formation of Si-N bonding) and others.

### 3.2. Co-Condensation

**Figure 16:**
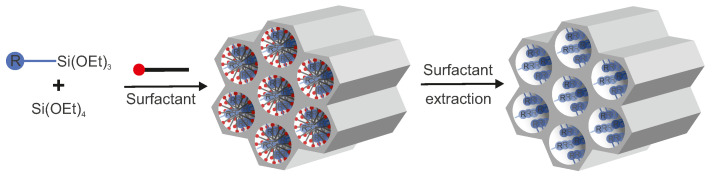
A general scheme of co-condensation.

As it was mentioned before, to form a mesoporous silica structure, it is necessary to have two primary reagents—a structure-directing agent (a surfactant) and a silica source (Figure 16).

Generally, only one surfactant and only one source of silica are used during the process. The most commonly used are tetraalkyl orthosilicate (TMOS, TEOS, TPOS or TBOS), fumed silica or a sodium silicate [104,105,106]. Nevertheless, using different combinations of both structure-directing agents and silica agents, at the same time, can be profitable giving brand new fields of opportunities for the researchers trying to tune properties of their materials. Different surfactants or their mixtures can strongly influence pore ordering or pore diameters in the three-dimensional structure of the material completely changing its character [107,108] while different silica sources impact the chemical properties and the composition of a final material.

It is possible to incorporate selected molecules during the formation of nanostructured silica in such a way that a proper functional unit will be present at the surface even after the removal of a surfactant. This method is called co-condensation (a one-pot synthesis or a direct synthesis) and, in most cases, depends on the condensation of tetraalkoxysilanes (RO)${}_{4}$Si (usually TEOS or TMOS) with one or more terminal trialkoxyorganosilanes (RO)${}_{3}$Si-R* in the presence of a corresponding surfactant. This approach uses the fact that due to the formation of the mesoporous structure, the obtained materials are characterized by a large surface-area-to-mass ratio. On the atomic level, it means that a large fraction of silicon-based groups remain unreacted after polycondensation (in the case of using only TEOS, its ≡SiOH, =Si(OH)${}_{2}$ and –Si(OH)${}_{3}$ groups). Therefore, it is not necessary that all silica “building blocks” form four bonds with other blocks in the matrix. In the case of a co-condensation with a mixture of (RO)${}_{4}$Si and (RO)${}_{3}$Si-R, the process leads to materials with organic R groups bound covalently to pore walls. Thus, additional -Si-R, =Si(OH)-R and –Si(OH)${}_{2}$-R groups are present. However, all this concerns only terminal organosilica precursors. For bridged-organosilanes, which have two trialkoxysilyl groups connected by a functional organic bridge, the process of polycondensation leads to the so-called Periodic Mesoporous Organosilicas (PMOs) (invented independently in 1999 by three research groups [109,110,111]) in which organic groups of an organic bridge are incorporated homogeneously in the pore walls of the obtained material. This topic is not covered in this article but is well described in the literature [112,113,114].

For trialkoxyorganosilanes, corresponding organic functions are mainly located at the surface of pores. This phenomenon can be explained by:

- the hydrophobicity of some organic R groups which tend to avoid interaction with polar silanol/siloxane groups;
- the hydrophobicity of organic residues; in some cases, they can interact with micelles. In this case, (RO)${}_{3}$Si-R* molecules act not only as silica agents but also as weak structure-deriving agents. Moreover, they often slightly change pore sizes in the obtained material. Additionally, the silica source and the main surfactant can also be in the form of a single molecule [114]. For example, cetyl triethoxysilane can also condense into a lamellar solid in a concentrated acidic solution without any other silica source or a surfactant. Both MCM-41 and MCM-48 can be synthesized by using n-tetradecyldimethy(3-trimethoxysilylpropyl)ammonium chloride as the covalently bound surfactant-silica source [115];
- in a basic medium, an organosilane component undergoes lower hydrolysis and at a decreased condensation rate, in comparison to the pure inorganic precursor, which leads to retarded participation in the polycondensation process [76].

Generally, this method has a few noticeable advantages in comparison with other methods:

- since it is a one-pot synthesis, this method saves time due to the decreased amount of stages in the synthesis route—the synthesis and functionalization occur at the same time;
- no material loss during the process of isolation and purification of the product between its synthesis and functionalization;
- condensation occurs at the same time with the 3D structure formation. Thus, pore blocking does not occur, and pores are filled with a surfactant. In contrast to this, in post-synthesis methods, when the concentration of trialkoxysilanes is too high and the time of reaction is too long, pores can be filled with a new formed silica-based substance and be blocked;
- generally, the organic functionalities are distributed more homogeneously than in materials synthesized with the grafting process.

As for the disadvantages of the method, we can list the following:

- The main problem with this method is related to the limitation of the maximal achievable density of the functional groups without the loss in the structure mesoscopic ordering. As it was mentioned before, not all silica agents need to have the possibility to form all four -Si-O-Si- bonds and some fraction of a silica agent can form only three (or less) bonds. However, in reality, when the amount of an agent, which cannot form all four bonds, increases, it starts to disrupt the mesoscale structure ordering. In practice, the content of organic functionalities in the modified silica phases does normally not exceed 40 molls, and typical values are even lower (5–15 molls).
- Due to the different hydrolysis and condensation rates, a part of the silica source and trialkoxysilanes can take part in homocondensation reactions and resulting in a lower number of bound organic groups.
- The increase in the concentration of a modification agent can lead to decreased pore diameter, pore volume, and specific surface area of the obtained mesoporous silica.
- Surfactants are often removed from the pores by calcination. However, this method cannot be used here since the process can destroy organic functional groups. Nevertheless, surfactants can be effectively removed by extractive methods.

### 3.3. Functional Groups and Their Reactions

In this section, we show a few examples of commonly used trialkoxyorganosilane reagents due to their common usage and practical benefits. Trialkoxyorganosilanes are readily available and, because of their trialkoxy-group, they can be readily and effectively chemically bound via the condensation process, both to a silica surface (grafting) and different silica-based molecules (co-condensation).

The chemistry of the synthesis and reactivity of trialkoxyorganosilanes is a separate topic, and herein we would like to show only prominent representatives of this group. In previous sections, we focused on various methods of the synthesis and showed how silica surface could be modified with the use of different classes of organic and silica-organic compounds. Below, we listed different functional groups which can be implemented on the surface of a modified material. Generally, this group can be divided into two groups—passive and reactive functional groups [75], as shown in Table 2.

In this context, passive functional groups characterized by low reactivity and alkyl chains or phenyl groups can be included here. Due to their low reactivity and high hydrophobicity, they are used to increase the hydrophobicity of a surface via the formation of the hydrocarbon-based hydrophobic layer. Here, the passivation of silanol groups occurs and, since the surface of silica is chemically bound and separated from the solution with a hydrophobic layer, the silica framework is protected from hydrolysis. Moreover, it has increased stability towards moisture and mechanical compression.

In contrast, active functional groups possess greater chemical reactivity, including the possibility of further functionalization, the formation of charged fragments, the adsorption of metal ions or different molecules, hydrogen bonding, etc. Thus, the materials functionalized with active functional groups can be applied in numerous fields such as precursors for novel materials, materials for medical application, stationary phases in chromatography, adsorbents for metal concentration and separation, catalysts and many others.

By using active functional groups as modifiers, instead of synthesizing several specific reagents with different functional groups, it is more convenient to use a small number of “standard” reagents for silica functionalization and subsequently perform reactions on materials modified by them [116]. Additional advantages of using those groups are high yields obtained under mild reaction conditions, the compatibility of different functional groups, no required catalytic activation and the absence of byproducts [117,118]. The following table (Table 3) include a short review of some of the possible reactions. For a more detailed look at those and other reactions, we recommend the source articles [77,116,119].

The last row of Table 3 shows the formation of salts with active functional groups. In this case, the ions of metals are immobilized. Those reactions can be extended to molecules which we will show in the next section.

Although active functional groups seem to be more beneficial in comparison with their passive equivalents, the latter can also play an important role. The passive functional units may be used in tuning the concentration of the active functional groups and, thus, adjust the concentration of our “2D solid solution”.

## 4. The Immobilization of Ions and Molecules by a 2D Solid Solvent: A Few Examples

In previous sections, we showed how to modify the surface of silica with functional units. We also presented what kind of functional molecules allow for homogenous immobilization of ions or molecules at a surface converting silica into a 2D solid solvent. Here, we show a few practical examples of such modifications along with experimental results confirming the success of the applied procedure. We also explain why specific matrix architectures were chosen for the purpose.

### 4.1. Grafting of Spherical Silica

Despite a relatively low specific surface area (in comparison to mesoporous silica) of approximately 13 m${}^{2}$/g [130], the spherical silica is vital for the immobilization of some molecules especially single-molecule magnets. In this case, the shape of the silica support is more critical than a specific surface area. Relatively small silica spheres can be precisely observed with the use of a TEM microscope. There are no hidden areas in this material (such as internal channels in mesoporous silica), therefore larger molecules (>1 nm) are visible at the surface of such a matrix, especially at the horizon of the sphere. Such a feature can be employed for direct observation of the immobilized molecules to gain knowledge about the attachment with no necessity of applying expensive and sophisticated techniques [131].

**Figure 17:**
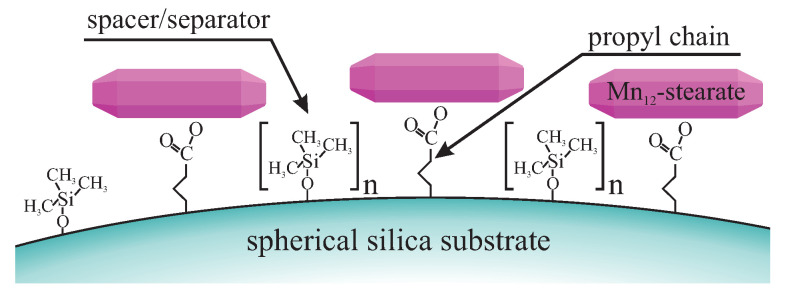
A schematic illustration of the spherical silica acting as a 2D solid solvent for the Mn${}_{12}$-stearate single-molecule magnets; n denotes the number of spacer units, separating single-molecule magnets.

The synthesis of soluble single-molecule magnets (SMMs)—Mn${}_{12}$-stearate was carried out according to the protocol shown by Park and his coworkers [132]. The spherical silica (prepared according to the optimized Stöber protocol—see: Supplementary Information) can be functionalized with propyl-carboxyl units to anchor the aforementioned magnetic molecules: stearic acids ligands readily react with a carboxylic acid resulting in a stearic acid molecule and Mn${}_{12}$-stearate bound *via* carboxyl group. It must be emphasized that such molecules can be attached via one, two, three or even four such bonds [133]. The modification of the surface of silica with proper anchoring units can be easily achieved by grafting method [130]. It would seem that a grafting method does not allow modifying the concentration of the functional units at the surface. However, this can be done with the use of the spacer units method [134,135]. A surface with anchoring units distributed with controlled statistical distances reflects the 2D solid solvent for Mn${}_{12}$-stearate SMMs since it can separate and keep them at the assumed concentration and distances from each other (see: Figure 17).

The procedure of the functionalization of silica involves the following steps (for details see Supplementary Information):

1. grafting the spherical silica by the precursors of anchoring (butyronitriletriethoxysilane—BNTES) and spacer (tetraethyl orthosilicate - TEOS) units: in this step, the statistical distances between anchoring units can be tuned by changing the proportion between TEOS and BNTES (defined by the *n* number in Figure 17);
2. the silanation of the surface hydroxyl units with the solution of chlorotrimethyl silane (ClTMS): this step is necessary to avoid unwanted side reactions between carboxylic acid groups and surface hydroxyl units during the hydrolysis;
3. acidic hydrolysis of cyano units into carboxylic acid groups.

**Figure 18:**
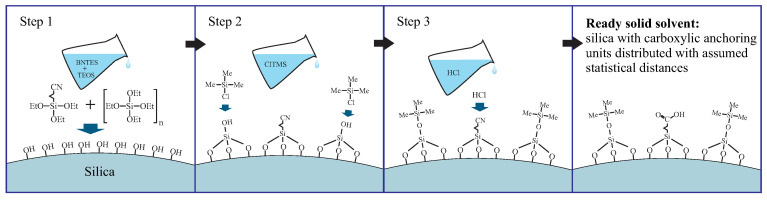
A schematic illustration of the synthesis of spherical silica containing carboxylic anchoring units distributed with the assumed statistical distances. Abbreviations: TEOS—tetraethyl orthosilicate, BNTES—butyronitriletriethoxysilane, ClTMS—chlorotrimethylsilane, Me—methyl groups, Et—ethyl units. The number *n* deneotes the density of anchoring units.

After the procedure, presented concisely in Figure 18, the functionalized silica can play a role of the 2D solid solvent. It can be used for materials such as silver ions or the aforementioned Mn${}_{12}$-stearate single-molecule magnets. Here, we present the latter example since it can be observed directly in a transmission electron microscope.

**Figure 19:**
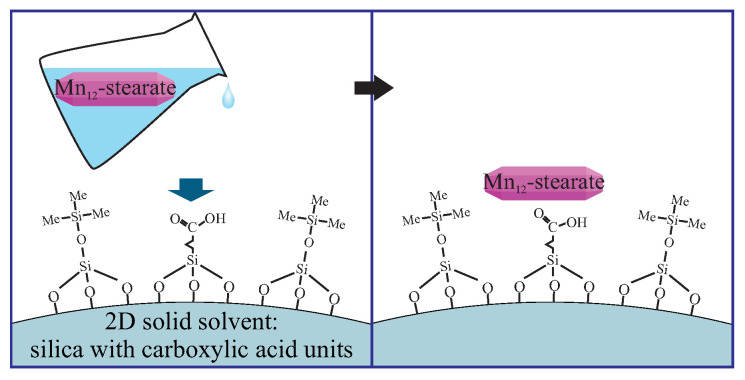
A schematic illustration of the immobilization of the Mn${}_{12}$-stearate magnetic molecules by functionalized silica.

The route is realized by the treatment of the functionalized silica by the solution of Mn${}_{12}$-stearate in dichloromethane, as shown in Figure 19. After this procedure, the anchoring units bond SMMs at the surface. The type of immobilization (the number of bonds between silica surface and SMMs) and the density of magnetic molecules depends only on the density of the anchoring units on the surface.

**Figure 20:**
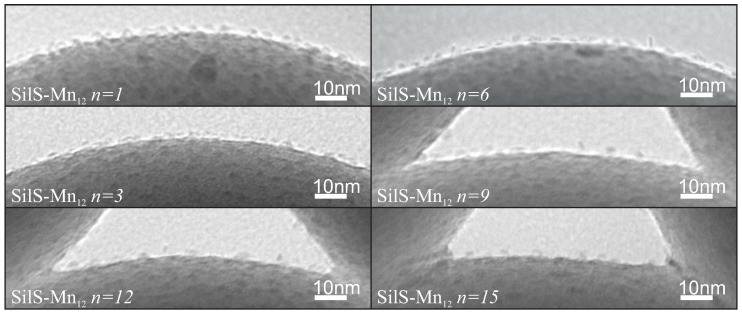
TEM micrographs of the Mn${}_{12}$-stearate molecules attached to the spherical silica-based materials containing various concentrations of the anchoring units separated by spacer groups. The *n* denotes the number of spacer units per single anchoring molecule.

The TEM micrograps (Figure 20) confirmed our assumptions:

- Mn${}_{12}$-stearate single-molecule magnets attached to the surface of silica can be directly observed with the use of TEM;
- we are able to tune the way of SMMs immobilization and their concentration at the matrix.

In this case, we observed an interesting dependency. At first glance, the concentration of SMMs does not change with an increasing number of spacer units up to six spacers per single anchoring unit (sample SilS-Mn${}_{12}$ *n* = 6). However, for larger amounts of spacer units, the concentration of the magnetic molecules significantly decreases. An Mn${}_{12}$-based molecule has 16 attaching points: eight at the circumference, four at the upper side and four at the bottom. As we proved earlier [130], Mn${}_{12}$-stearate molecules attach in an umbrella-like configuration to the surface and, thus, SMMs can be attached *via* 1–4 points. With the decreasing density of anchoring points, we decrease the number of bonds between the surface and SMMS. Next, when the density of anchors is sufficiently low, the density of anchored magnetic molecules decreases as well. The additional confirmation of this finding and a quantitative analysis of the number of anchored molecules at the silica was obtained by means of differential pulse anodic stripping voltammetry (DPASV) and was in great accordance with our assumptions. It is important to note, that the number of bonds between the silica and SMMs also may influence on their magnetic properties [133].

We confirmed that the spherical silica with carboxylic acid groups separated by spacers can play an essential role of an efficient 2D solid solvent for Mn${}_{12}$-based molecules. In this case, we can tune not only the concentration of the immobilized molecules but also the type of their immobilization. Moreover, due to the thermal and mechanical stability of silica substrate there is a possibility for study of magnetic ageing effects of deposited SMMs [136].

In this place, one can ask about the applicative potential of such a solution. However, the technology of the controlled grafting of Mn${}_{12}$ -stearate molecules in combination with the procedure of preparing vertically aligned porous silica thin films functionalized at the pores bottom [137] can result in the system of molecular neurons [138,139] or a super-dense magnetic memory storage [140]. Thus, the approach presented here can be essential for emerging technologies.

### 4.2. Mesoporous Silica for the Immobilization of Metal Ions and Molecules

The SBA-15 silica is an excellent candidate for a matrix in our 2D solid solvent concept. As it was mentioned in previous sections, 2D hexagonally distributed channels result in an enormous specific surface area. Over 700 m${}^{2}$/g is available for a homogenous distribution of the ions or molecules. This feature gives to the functionalized SBA-15 silica huge applicative potential as an absorbent for heavy metals in natural environment. The SBA-15 silica with proper anchoring units can be pressed into some kind of removable cartridge (pellet) and be a kind of filter/absorber for remediation of the groundwater or soil. The immobilization of metals can do it in a given place of the environment, i.e., transformation into insoluble or sparingly soluble compounds, making it less available or unavailable to plants, which contributes to for their further migration into the soil profile [141]. Such a cartridge can be later removed from the soil and replaced to another one.

The synthesis procedure enables both grafting and a direct synthesis to attach surface anchoring units. In this case, the co-condensation method seems to be more profitable because of a homogenous distribution of functional units and the lack of pore blocking.

**Figure 21:**
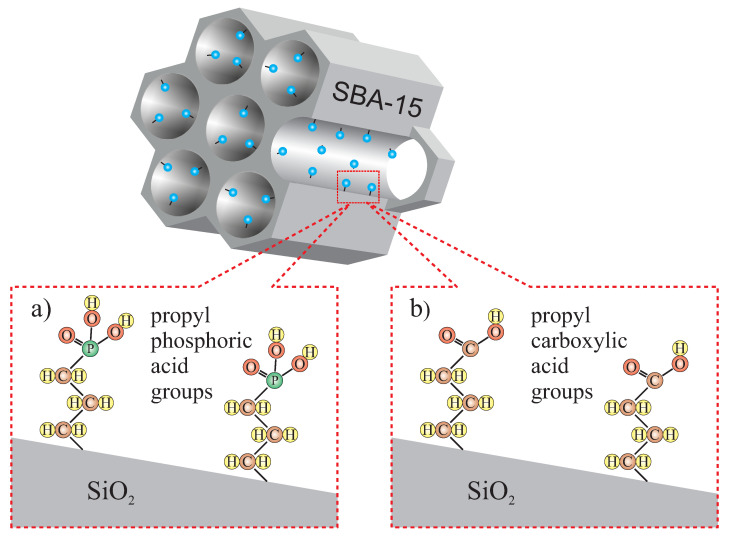
A schematic presentation of the SBA-15 silica functionalized with propyl phosphoric acid (**a**) and propyl carboxylic acid groups (**b**).

In this section, we present the SBA-15 silica containing propyl phosphonate and propyl carboxylic acid groups (presented schematically in Figure 21). Both functional molecules can be attached in co-condensation of TEOS with various precursors in the presence of P123 surfactant. Both materials can be used as a 2D solid solvent for different ions and molecules.

**Figure 22:**
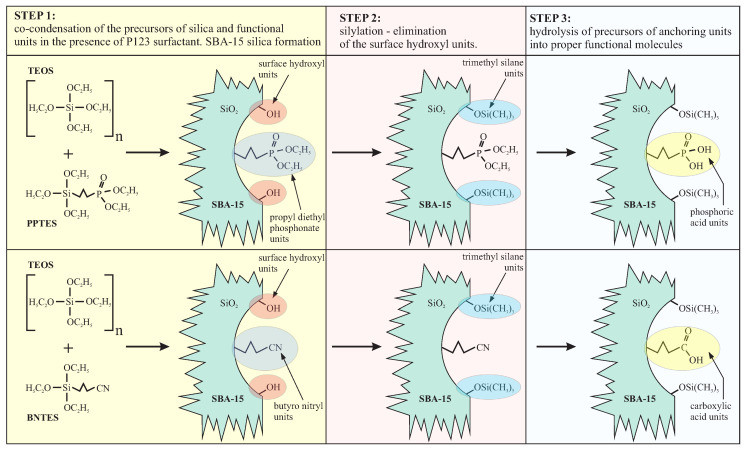
A diagram of the routes of the syntheses of the SBA-15 silica containing anchoring units: a propyl phosphoric acid (upper route) and a propyl carboxylic acid (bottom route). The number *n* denotes molls the number of tetraethyl orthosilicate (TEOS) per single moll of the precursor of functional units (phosphonate propyl diethyltriethoxysilane – PPTES or butyronitriletriethoxysilane—BNTES depending on the desired material).

The synthesis of both materials is similar, as presented in Figure 22. In the first step, the co-condensation of tetraethyl orthosilicate (TEOS) with precursors of anchoring units (phosphonate propyl diethyl triethoxysilane—PPTES for the silica containing phosphoric acid or BNTES for SBA-15 with carboxylic acid units) is performed in the presence of triblock copolymer Pluronic P123 ($EO_{20}PO_{70}EO_{20}$, where EO poly-ethylene oxide and PO is poly-propylene oxide) as the structuring agent in acidic conditions. The final concentration of the functional units can be adjusted by changing the proportions of the precursor of silica (TEOS) and the precursors of functional units (PPTES or BNTES, depending on the aimed material). These proportions can be set by *n* number describing the molls number of tetraethyl orthosilicate per single moll of a precursor of functional units. In the next step, surface hydroxyl units are eliminated by silylation. Finally, the anchoring of the units is performed by hydrolysis. The detailed description of syntheses can be found in Supplementary Materials.

The SBA-15 with propyl phosphoric acid units can be applied as a 2D solid solvent for two-valence metal ions such as copper, nickel, iron or cobalt [126,127,142]. However, we must remark that it is difficult to confirm the presence of homogeneously distributed anchored ions. Nevertheless, we proposed an efficient procedure based on the vibrational spectroscopy supported by numerical simulations [128].

Ions dissolved inside a silica matrix can be applied in photonics: by choosing a particular concentration of ions, it is possible to tune the nonlinear optical response of such a material [143]. Powders containing copper ions distributed at deficient concentrations are efficient biocidal material [144].

Such materials can also act as a starting point for the preparation of nanocomposites containing nanocrystals inside silica pores. Those crystals can be treated as quantum dots [145,146]. In this case, a proper “dilution” of metal ions inside a matrix is crucial to obtain the assumed final effect.

The SBA-15 with propyl carboxylic acid units can efficiently dilute single-valent ions (such as silver) and magnetic molecules (like various derivatives of Mn${}_{12}$) on its surface. Attaching magnetic molecules based on Mn${}_{12}$ at specific concentrations and distances between each other, enables the investigation of the properties of separated single-molecule magnets [129,147] which is a no small achievement. However, using our proposed 2D solid solvent, it is achievable by a simple wet-chemistry process by correctly setting of the dilution rate, and adjusting the proportions between precursors (number *n* in Figure 22). Dulski and others [148] presented the immobilization of silver ions by SBA-15 silica and used the material as an antimicrobial agent. Moreover, the calcination of such material resulted in a novel silica-based nanocomposite containing silver or silver oxide nanocrystals inside pores.

All of the aforementioned materials owe their properties to treating the functionalized SBA-15 silica as a 2D solid solvent and the possibility of adjusting the dilution rate of the incorporated species.

### 4.3. Mesoporous Silica Functionalized with Cyclam for Chelating of Chlorides

**Figure 23:**
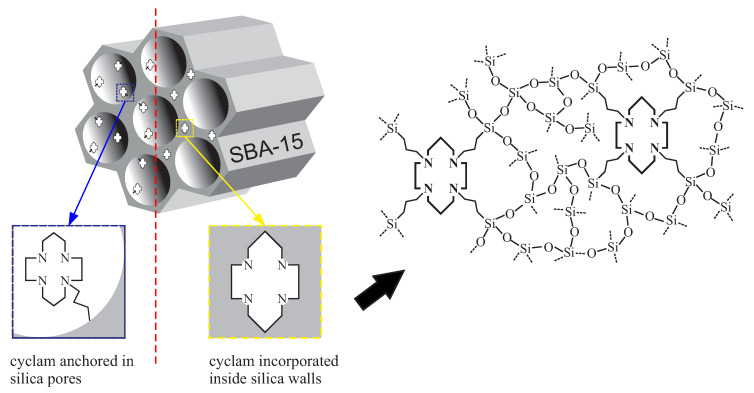
A diagram of the structure of silica modified by cyclam (1,4,8,11-Tetraazacyclotetradecane) molecules: anchored inside silica pores (left side) and incorporated inside the walls along with the schematic molecular structure (right side—dashed lines represent the continuation of the structure).

The cyclam (1,4,8,11-Tetraazacyclotetradecane) is a strongly chelating molecule which can be incorporated in the SBA-15 silica. Interestingly, such molecule can either be attached inside silica pores or built inside silica walls, as presented in Figure 23.

As with the previously presented material, also in this case, one can notice the tremendous applicative potential in water and soil remediation. In this case, however, the material can absorb chlorides of heavy metals—this function seems to be complementary to the case presented in the previous section. Due to the possibility for functionalization inside pores and in the walls structure, one can obtain great capacity for chelation of chlorides. It is possible to obtain the material possessing functional units inside channels and walls at the same time.

**Figure 24:**
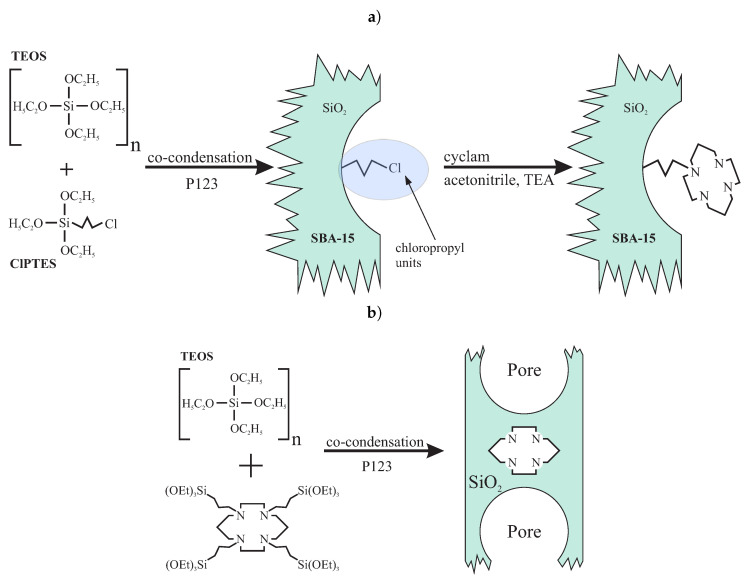
The synthesis routes for the SBA-15 silica containing cyclam molecules attached inside silica pores *via* propyl chains (**a**) and incorporated inside the structure of silica (**b**).

Despite the same functional molecule, the synthesis routes for both types of the materials are different. For cyclam inside the pores of the SBA-15, the procedure consists of a few steps: firstly, the co-condensation of TEOS and chloropropyl triethylsilane (ClPTES) is performed in acidic conditions in the presence of the P123 surfactant. In the next step, the chlorine is substituted by cyclam dissolved in acetonitrile and triethylamine (TEA). The SBA-15 containing cyclam incorporated in silica walls is fabricated in a one-step reaction: the co-condensation of TEOS with tetrasilylated cyclam (1,4,8,11 trietoxysilane propyl 1,4,8,11-tetraazacyclotetradecane). Both reactions are presented in Figure 24. In both cases, the concentration of cyclam units can be finely adjusted by the modification of the proportions between precursors of silica and cyclam during co-condensation (the *n* number describing the number of the molls of tetraethyl orthosilicate per a single moll of the precursor of functional units). A detailed description of syntheses can be found in Supplementary Materials.

Both kinds of the material can play the role of a 2D solvent for the same molecules: metal (II) chlorides (copper, nickel, iron, or cobalt). Chlorides are chelated by cyclam molecules and are immobilized in this form. The efficiency of this process was unambiguously confirmed by the electron paramagnetic resonance (EPR) technique. For both materials treated by nickel chloride, the super hyperfine coupling was observed. This phenomenon proved that nickel chlorides are separated from each other and regularly distributed inside pores [149] and within the walls of silica structure [150]. Considering the importance of such materials, we can quote environmental protection and recycling. Silica is insoluble in most solvents (except for the extreme solvent, a hydrofluoric acid, and strong bases). Thus, it can be used as an adsorber for chlorides from polluted water which can then be removed by filtration. Because of the large specific surface area, further extended by the possibility of functionalization inside walls, its capacity to adsorb chlorides is enormous. Thus, efficiency is expected to be high. Again, in this case, the 2D solid solvent concept resulted in the fabrication of a functional nanomaterial with high applicative potential.

## 5. Conclusions

In this work, we proposed the concept of a 2D solid solvent—a novel class of materials to capture and separation of ions or molecules. Such materials can be fabricated based on functionalized nanostructured silica and used to immobilize at the surface particular species at a specific concentration with the desired distance from each other. We reviewed its fabrication process, possible applications and explained the way of incorporating selected molecules and ions in its volume or surface.

Indeed, in some cases, a similar effect can be achieved by other techniques. However, the procedure to capture of ions or molecules by the 2D solid solvent proposed here in numerous examples is easy to apply, time-efficient and reliable (in terms of repeatability). Moreover, most of the steps of the process and required reagents proposed in this focused review are low-cost and efficient. This research field is open for new investigations with numerous horizons of applicability. We believe that this review can be of particular aid for researchers wanting to add this promising class of materials to their research repertoire.

## Figures and Tables

**Table 1:** Example types of mesoporous materials with different interactions between the surfactant and the inorganic framework. (Notation: *S*, *N*—a surfactant, *I*—an inorganic precursor, *X*—a counter ion).

Mesophase (Structure)	Surfactant	Interaction Pathway	Interaction Scheme
MCM-41 (2D hexadonal *P6mm*), MCM-48 (3D cubic *Ia3d*), FSM-16 (2D hexagonal *P6mm*)	CTAB (cetyltrimethylammonium bromide)	$S^{+}I^{-}$ (electrostatic force)	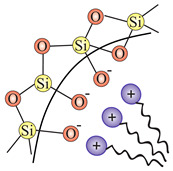
SBA-1 (Cubic *Pm3n*), SBA-2 (3D hexagonal *P6${}_{3}$/mmc*)	GS (gemini surfactant)	$S^{+}$$X^{-}$$I^{+}$ (electrostatic force)	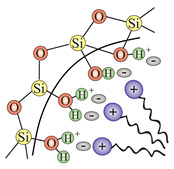
SBA-3 (2D hexagonal *P6mm*)	CTAB		
HMS (hexagonal-like)	DDA (dodecylamine)	$S^{0}$$I^{0}$ (hydrogen bond)	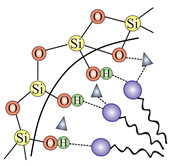
MSU (hexagonal-like)	PEO (poly(ethylene oxide)-based)	$N^{0}$$I^{0}$ (hydrogen bond)	
SBA-15 (2D hexagonal *P6mm*)	Pluronic P123	$\left(S^{0}H^{+}\right)X^{-}I^{+}$ (weak electrostatic force)	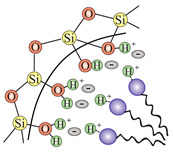

**Table 2:** The most used trialkoxyorganosilanes for silica surface functioanalization.

Class	Formula	Substituent
Passive functional groups		
Alkanes	(RO)${}_{3}$Si-CH${}_{3}$	methyl
	(RO)${}_{3}$Si-C${}_{2}$H${}_{5}$	ethyl-
	(RO)${}_{3}$Si-C${}_{3}$H${}_{7}$	propyl-
	(RO)${}_{3}$Si-CH${}_{2}$-CH-(CH${}_{3}$)${}_{2}$	isobutyl-
	(RO)${}_{3}$Si-C${}_{8}$H${}_{17}$	octyl-
	(RO)${}_{3}$Si-C${}_{12}$H${}_{25}$	dodecyl-
Fluorinated alkanes	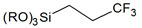	3,3,3-trifluoropropyl-
	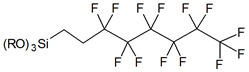	1H,1H,2H,2H-perfluorooctyl-
Cycloalkanes	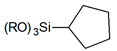	cyclopentyl-
	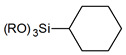	cyclohextyl-
Aromatic rings	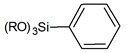	phenyl-
	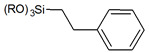	2-phenylethyl-
Reactive functional groups		
Alkenes	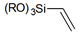	vinyl-
	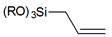	allyl-
	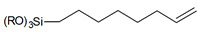	7-octen-1-yl-
Halogen-based	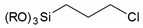	3-chloropropyl-
	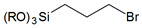	3-bromopropyl-
	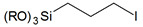	3-iodopropyl-
Simple terminal heterogroups	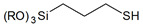	3-mercaptopropyl-
	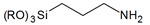	3-aminopropyl-
	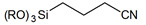	3-(Triethoxysilyl)propionitrile
	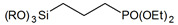	1
Complex terminal organics	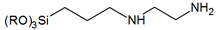	N-ethylenediamino-
	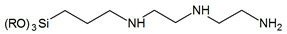	N-diethylenetriamino-
	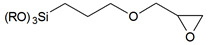	3-glycidyloxypropyl-
	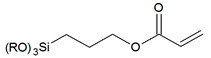	3-propyl acrylate

**Table 3:** Selected reactions on different functional groups.

Reaction Type	Reaction	Reference
Nucleophilic substitution	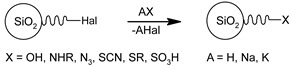	[77,117]
“Click” alkyne-azide cycloaddition	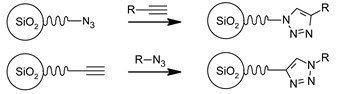	[118,120]
Thiol-Ene “click”	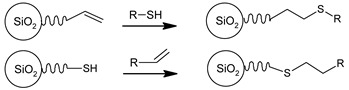	[121,122]
Oxidation	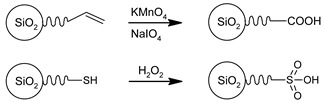	[123,124,125]
Hydrolysys	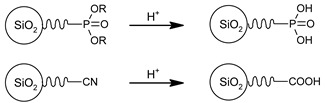	[126,127]
Salt formation	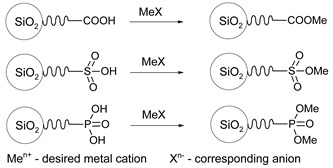	[128,129]

## Supplementary Materials

The following are available online at https://www.mdpi.com/1422-0067/21/21/8137/s1.

## Abbreviations

The following abbreviations are used in this manuscript:

TEOS	tetraethyl orthosilicate
TMOS	tetramethyl orthosilicate
TPOS	tetrapentyl orthosilicate
TBOS	tetrabutyl orthosilicate
PPTES	phosphonate propyl diethyl triethoxysilane
BNTES	butyronitrile triethoxysilane / cyanopropyl triethoxysilane
P123	(poly(ethylene glycol)${}_{20}$-poly(propylene glycol)${}_{70}$-poly(ethylene glycol)${}_{20}$
CTAB	cetyl triethyl ammonium bromide

## References

[1] Feynman R.P. (2012). There’s plenty of room at the bottom: An invitation to enter a new field of physics. Handbook of Nanoscience, Engineering, and Technology.

[2] Corriu R., Mehdi A., Reyé C. (2004). Nanoporous materials: A good opportunity for nanosciences. J. Organomet. Chem..

[3] Guo Z., Tan L. (2009). Fundamentals and Applications of Nanomaterials.

[4] Hussain S.A., Dey B., Bhattacharjee D., Mehta N. (2018). Unique supramolecular assembly through Langmuir–Blodgett (LB) technique. Heliyon.

[5] Basu J., Sanyal M. (2002). Ordering and growth of Langmuir–Blodgett films: X-ray scattering studies. Phys. Rep..

[6] Mei Q.X., Lai L., Li S.J., Mei P., Wang Y.Q., Ma Q.L., Liu Y. (2019). Surface properties and phase behavior of Gemini/conventional surfactant mixtures based on multiple quaternary ammonium salts. J. Mol. Liq..

[7] (2019). Mixed crystal (solid solution). IUPAC Compendium of Chemical Terminology.

[8] Holt P., King D. (1955). The chemistry of silica surfaces. J. Chem. Soc. (Resumed).

[9] Bergna H.E. (1994). Colloid Chemistry of Silica: An Overview.

[10] Iler R.K., Iler R. (1979). The chemistry of silica: Solubility, polymerization, colloid and surface properties, and biochemistry. Sci. Geol. Bull. Mem..

[11] Stöber W., Fink A., Bohn E. (1968). Controlled growth of monodisperse silica spheres in the micron size range. J. Colloid Interface Sci..

[12] Kim T.G., An G.S., Han J.S., Hur J.U., Park B.G., Choi S.C. (2017). Synthesis of size controlled spherical silica nanoparticles via sol-gel process within hydrophilic solvent. J. Korean Ceram. Soc..

[13] Ibrahim I.A., Zikry A., Sharaf M.A. (2010). Preparation of spherical silica nanoparticles: Stober silica. J. Am. Sci..

[14] Wang X.D., Shen Z.X., Sang T., Cheng X.B., Li M.F., Chen L.Y., Wang Z.S. (2010). Preparation of spherical silica particles by Stöber process with high concentration of tetra-ethyl-orthosilicate. J. Colloid Interface Sci..

[15] Kolbe G. (1956). Das Komplexchemische Verhalten der Kieselsäure. Ph.D. Thesis.

[16] Bogush G., Tracy M., Zukoski Iv C. (1988). Preparation of monodisperse silica particles: Control of size and mass fraction. J. Non-Cryst. Solids.

[17] Chang S.M., Lee M., Kim W.S. (2005). Preparation of large monodispersed spherical silica particles using seed particle growth. J. Colloid Interface Sci..

[18] Enomoto N., Koyano T., Nakagawa Z.E. (1996). Effect of ultrasound on synthesis of spherical silica. Ultrason. Sonochem..

[19] Matsoukas T., Gulari E. (1988). Dynamics of growth of silica particles from ammonia-catalyzed hydrolysis of tetra-ethyl-orthosilicate. J. Colloid Interface Sci..

[20] Matsoukas T., Gulari E. (1989). Monomer-addition growth with a slow initiation step: A growth model for silica particles from alkoxides. J. Colloid Interface Sci..

[21] Napper D.H. (1970). Colloid stability. Ind. Eng. Chem. Prod. Res. Dev..

[22] Bogush G., Zukoski Iv C. (1991). Studies of the kinetics of the precipitation of uniform silica particles through the hydrolysis and condensation of silicon alkoxides. J. Colloid Interface Sci..

[23] Bogush G., Zukoski Iv C. (1991). Uniform silica particle precipitation: An aggregative growth model. J. Colloid Interface Sci..

[24] Wells J., Koopal L., De Keizer A. (2000). Monodisperse, nonporous, spherical silica particles. Colloids Surf. A Physicochem. Eng. Asp..

[25] Van Blaaderen A., Kentgens A. (1992). Particle morphology and chemical microstructure of colloidal silica spheres made from alkoxysilanes. J. Non-Cryst. Solids.

[26] Van Blaaderen A., Van Geest J., Vrij A. (1992). Monodisperse colloidal silica spheres from tetraalkoxysilanes: Particle formation and growth mechanism. J. Colioid Interface Sci..

[27] Masalov V., Sukhinina N., Kudrenko E., Emelchenko G. (2011). Mechanism of formation and nanostructure of Stöber silica particles. Nanotechnology.

[28] Karpov I., Samarov E., Masalov V., Bozhko S., Emelchenko G. (2005). The intrinsic structure of spherical particles of opal. Phys. Solid State.

[29] Samarov E., Mokrushin A., Masalov V., Abrosimova G., Emelchenko G. (2006). Structural modification of synthetic opals during thermal treatment. Phys. Solid State.

[30] Van Helden A., Jansen J., Vrij A. (1981). Preparation and characterization of spherical monodisperse silica dispersions in nonaqueous solvents. J. Colloid Interface Sci..

[31] Rouquerol J., Fairbridge C., Everett D., Haynes J., Pernicone N., Ramsay J., Sing K., Unger K. (1994). Recommendations for the Characterization of Porous Solids. Pure Appl. Chem..

[32] Cronstedt A.F. (1756). Svenska Vetenskaps Akademiens Handlingar Stockholm. Nat. Zeolite Miner..

[33] Chiola V., Ritsko J.E., Vanderpool C.D. (1971). Process for Producing Low Bulk Density Silica. U.S. Patent.

[34] DiRenzo F., Cambon H., Dutartre R. (1997). A 28-year-old synthesis of micelle-templated mesoporous silica. Microporous Mater..

[35] Yanagisawa T., Shimizu T., Kuroda K., Kato C. (1990). The Preparation of Alkyltriinethylaininonium–Kaneinite Complexes and Their Conversion to Microporous Materials. Bull. Chem. Soc. Jpn..

[36] Inagaki S., Fukushima Y., Kuroda K. (1993). Synthesis of highly ordered mesoporous materials from a layered polysilicate. Chem. Commun..

[37] Inagaki S., Terasaki O. (2004). FSM-16 and mesoporous organosilicas. Mesoporous Crystals and Related Nano-Structured Materials.

[38] Kresge C., Leonowicz M., Roth W., Vartuli J., Beck J. (1992). Ordered mesoporous molecular sieves synthesized by a liquid-crystal template mechanism. Nature.

[39] Beck J.S., Vartuli J.C., Roth W.J., Leonowicz M.E., Kresge C.T., Schmitt K.D., Chu C.T.W., Olson D.H., Sheppard E.W., McCullen S.B. (1992). A new family of mesoporous molecular sieves prepared with liquid crystal templates. J. Am. Chem. Soc..

[40] Tang F., Li L., Chen D. (2012). Mesoporous silica nanoparticles: Synthesis, biocompatibility and drug delivery. Adv. Mater..

[41] Galarneau A., Nader M., Guenneau F., Di Renzo F., Gedeon A. (2007). Understanding the stability in water of mesoporous SBA-15 and MCM-41. J. Phys. Chem. C.

[42] Tanev P.T., Pinnavaia T.J. (1996). Biomimetic Templating of Porous Lamellar Silicas by Vesicular Surfactant Assemblies. Science.

[43] Bagshaw S.A., Prouzet E., Pinnavaia T.J. (1995). Templating of Mesoporous Molecular Sieves by Nonionic Polyethylene Oxide Surfactants. Science.

[44] Zhao D., Feng J., Huo Q., Melosh N., Fredrickson G.H., Chmelka B.F., Stucky G.D. (1998). Triblock Copolymer Syntheses of Mesoporous Silica with Periodic 50 to 300 Angstrom Pores. Science.

[45] Wanka G., Hoffmann H., Ulbricht W. (1994). Phase diagrams and aggregation behavior of poly (oxyethylene)-poly (oxypropylene)-poly (oxyethylene) triblock copolymers in aqueous solutions. Macromolecules.

[46] Celer E.B., Kruk M., Zuzek Y., Jaroniec M. (2006). Hydrothermal stability of SBA-15 and related ordered mesoporous silicas with plugged pores. J. Mater. Chem..

[47] Attard G.S., Glyde J.C., Goltner C.G. (1995). Liquid-Crystalline Phases as Templates for the Synthesis of Mesoporous Silica. Nature.

[48] Chen C.Y., Burkett S.L., Li H.X., Davis M.E. (1993). Studies on mesoporous materials II. Synthesis mechanism of MCM-41. Microporous Mater..

[49] Huo Q., Margolese D.I., Ciesla U., Demuth D.G., Feng P., Gier T.E., Sieger P., Firouzi A., Chmelka B.F. (1994). Organization of Organic Molecules with Inorganic Molecular Species into Nanocomposite Biphase Arrays. Chem. Mater..

[50] Rosen M., Kunjappu J. (2012). Surfactants and Interfacial Phenomena.

[51] Vert M., Doi Y., Hellwich K.H., Hess M., Hodge P., Kubisa P., Rinaudo M., Schué F. (2012). Terminology for biorelated polymers and applications (IUPAC Recommendations 2012). Pure Appl. Chem..

[52] Hunter R. (1987). Foundations of Colloid Science.

[53] Lawrence M.J. (1994). Surfactant systems: Their use in drug delivery. Chem. Soc. Rev..

[54] Klabunde K., Richards R. (2009). Nanoscale Materials in Chemistry.

[55] Huo Q., Margolese D., Ciesla U.E.A. (1994). Generalized synthesis of periodic surfactant/inorganic composite materials. Nature.

[56] Firouzi A., Kumar D., Bull L., Besier T., Sieger P., Huo Q., Walker S., Zasadzinski J., Glinka C., Nicol J. (1995). Cooperative organization of inorganic-surfactant and biomimetic assemblies. Science.

[57] Choi D.G., Yang S.M. (2003). Effect of two-step sol-gel reaction on the mesoporous silica structure. J. Colloid Interface Sci..

[58] Alexandridis P., Hatton T.A. (1995). Poly(ethylene oxide)–poly(propylene oxide)–poly(ethylene oxide) block copolymer surfactants in aqueous solutions and at interfaces: Thermodynamics, structure, dynamics, and modeling. Colloids Surf. A Physicochem. Eng. Asp..

[59] De Paul S.M., Zwanziger J.W., Ulrich R., Wiesner U., Spiess H.W. (1999). Structure, Mobility, and Interface Characterization of Self-Organized Organic-Inorganic Hybrid Materials by Solid-State NMR. J. Am. Chem. Soc..

[60] Kiselev A.V., Lygin V.I. (1975). Infrared Spectra of Surface Compounds.

[61] Li J., Yang C., Zhang Q., Li Z., Huang W. (2015). Effects of Fe addition on the structure and catalytic performance of mesoporous Mn/Al–SBA-15 catalysts for the reduction of NO with ammonia. Catal. Commun..

[62] Kim J., Ichikuni N., Hara T., Shimazu S. (2016). Study on the selectivity of propane photo-oxidation reaction on SBA-15 supported Mo oxide catalyst. Catal. Today.

[63] Guan M., Liu W., Shao Y., Huang H., Zhang H. (2009). Preparation, characterization and adsorption properties studies of 3-(methacryloyloxy) propyltrimethoxysilane modified and polymerized sol–gel mesoporous SBA-15 silica molecular sieves. Microporous Mesoporous Mater..

[64] dos Santos S.M.L., Nogueira K.A.B., de Souza Gama M., Lima J.D.F., da Silva Júnior I.J., de Azevedo D.C.S. (2013). Synthesis and characterization of ordered mesoporous silica (SBA-15 and SBA-16) for adsorption of biomolecules. Microporous Mesoporous Mater..

[65] Vansant E., Van Der Voort P., Vrancken K. (1995). Characterization and Chemical Modification of the Silica Surface.

[66] Ek S., Root A., Peussa M., Niinistö L. (2001). Determination of the hydroxyl group content in silica by thermogravimetry and a comparison with 1H MAS NMR results. Thermochim. Acta.

[67] McCool B., Murphy L., Tripp C.P. (2006). A simple FTIR technique for estimating the surface area of silica powders and films. J. Colloid Interface Sci..

[68] Zholobenko V.L., Plant D., Evans A.J., Holmes S.M. (2001). Acid sites in mesoporous materials: A DRIFTS study. Microporous Mesoporous Mater..

[69] Leonardelli S., Facchini L., Fretigny C., Tougne P., Legrand A.P. (1992). Silicon-29 NMR study of silica. J. Am. Chem. Soc..

[70] Liu C.C., Maciel G.E. (1996). The Fumed Silica Surface: A Study by NMR. J. Am. Chem. Soc..

[71] Delitala C., Cadoni E., Delpiano D., Meloni D., Alba M., Becerro A., Ferino I. (2009). Liquid-phase thiophene adsorption on MCM-22 zeolites. Acidity, adsorption behaviour and nature of the adsorbed products. Microporous Mesoporous Mater..

[72] Chen J., Li Q., Xu R., Xiao F. (1996). Distinguishing the Silanol Groups in the Mesoporous Molecular Sieve MCM-41. Angew. Chem. Int. Ed. Engl..

[73] Zhao X.S., Lu G.Q., Whittaker A.K., Millar G.J., Zhu H.Y. (1997). Comprehensive Study of Surface Chemistry of MCM-41 Using 29Si CP/MAS NMR, FTIR, Pyridine-TPD, and TGA. J. Phys. Chem. B.

[74] Ide M., El-Roz M., De Canck E., Vicente A., Planckaert T., Bogaerts T., Van Driessche I., Lynen F., Van Speybroeck V., Thybault-Starzyk F. (2013). Quantification of silanol sites for the most common mesoporous ordered silicas and organosilicas: Total versus accessible silanols. Phys. Chem. Chem. Phys..

[75] Stein A., Melde B.J., Schroden R.C. (2000). Hybrid Inorganic-Organic Mesoporous Silicates—Nanoscopic Reactors Coming of Age. Adv. Mater..

[76] Hoffmann F., Fröba M. (2011). Vitalising porous inorganic silica networks with organic functions—PMOs and related hybrid materials. Chem. Soc. Rev..

[77] Herzer N., Hoeppener S., Schubert U.S. (2010). Fabrication of patterned silane based self-assembled monolayers by photolithography and surface reactions on silicon-oxide substrates. Chem. Commun..

[78] Zapilko C., Widenmeyer M., Nagl I., Estler F., Anwander R., Raudaschl-Sieber G., Groeger O., Engelhardt G. (2006). Advanced Surface Functionalization of Periodic Mesoporous Silica: Kinetic Control by Trisilazane Reagents. J. Am. Chem. Soc..

[79] Shimada T., Aoki K., Shinoda Y., Nakamura T., Tokunaga N., Inagaki S., Hayashi T. (2003). Functionalization on Silica Gel with Allylsilanes. A New Method of Covalent Attachment of Organic Functional Groups on Silica Gel. J. Am. Chem. Soc..

[80] Yeon Y.R., Park Y., Lee J.S., Park J.W., Kang S.G., Jun C.H. (2008). Sc(OTf)3-Mediated Silylation of Hydroxy Functional Groups on a Solid Surface: A Catalytic Grafting Method Operating at Room Temperature. Angew. Chem. Int. Ed..

[81] Fukaya N., Haga H., Tsuchimoto T., ya Onozawa S., Sakakura T., Yasuda H. (2010). Organic functionalization of the surface of silica with arylsilanes. A new method for synthesizing organic–inorganic hybrid materials. J. Organomet. Chem..

[82] Park J.W., Park Y.J., Jun C.H. (2011). Post-grafting of silica surfaces with pre-functionalized organosilanes: New synthetic equivalents of conventional trialkoxysilanes. Chem. Commun..

[83] Pujari S.P., Scheres L., Marcelis A.T.M., Zuilhof H. (2014). Covalent Surface Modification of Oxide Surfaces. Angew. Chem. Int. Ed..

[84] Kang H.J.H., Ali R.F., Paul M.T.Y., Radford M.J., Andreu I., Lee A.W.H., Gates B.D. (2019). Tunable functionalization of silica coated iron oxide nanoparticles achieved through a silanol–alcohol condensation reaction. Chem. Commun..

[85] Halász I., Sebestian I. (1969). New Stationary Phase for Chromatography. Angew. Chem. Int. Ed. Engl..

[86] Ter Maat J., Regeling R., Yang M., Mullings M.N., Bent S.F., Zuilhof H. (2009). Photochemical covalent attachment of alkene-derived monolayers onto hydroxyl-terminated silica. Langmuir.

[87] Taghizadeh M.J., Afghihi S., Saidi H. (2018). Superhydrophobic surface based silica nanoparticle modified with diisocyanate and short and long normal chain alcohols. Asian J. Nanosci. Mater..

[88] Stapleton J.J., Suchy D.L., Banerjee J., Mueller K.T., Pantano C.G. (2010). Adsorption Reactions of Carboxylic Acid Functional Groups on Sodium Aluminoborosilicate Glass Fiber Surfaces. ACS Appl. Mater. Interfaces.

[89] Kamitori Y., Hojo M., Masuda R., Kimura T., Yoshida T. (1986). Selective protection of carbonyl compounds. Silica gel treated with thionyl chloride as an effective catalyst for thioacetalization. J. Org. Chem..

[90] Clark J.H., Williamson C.J. (1993). Formation of Si—C bonds at the surface of silica and glass fibres. J. Mater. Chem..

[91] Maity N., Barman S., Abou-Hamad E., D’Elia V., Basset J.M. (2018). Clean chlorination of silica surfaces by a single-site substitution approach. Dalton Trans..

[92] Locke D.C., Schmermund J.T., Banner B. (1972). Bonded stationary phases for chromatography. Anal. Chem..

[93] Unger K., Thomas W., Adrian P. (1973). Herstellung oberflächenmodifizierter Adsorbentien. Kolloid-Z. Z. für Polym..

[94] Brust O.E., Sebestian I., Halász I. (1973). Stationäre phasen mit S-N bindung für die flügkeitschromatographie. J. Chromatogr. A.

[95] Price P.M., Clark J.H., Macquarrie D.J. (2000). Modified silicas for clean technology. J. Chem. Soc. Dalton Trans..

[96] Dash S., Mishra S., Patel S., Mishra B.K. (2008). Organically modified silica: Synthesis and applications due to its surface interaction with organic molecules. Adv. Colloid Interface Sci..

[97] Borges E.M. (2014). Silica, Hybrid Silica, Hydride Silica and Non-Silica Stationary Phases for Liquid Chromatography. J. Chromatogr. Sci..

[98] Pesek J.J., Matyska M.T. (1997). Methods for the Modification and Characterization of Oxide Surfaces. Interface Sci..

[99] Pesek J.J., Matyska M.T. (2009). Our favorite materials: Silica hydride stationary phases. J. Sep. Sci..

[100] Sandoval J.E., Pesek J.J. (1989). Synthesis and characterization of a hydride-modified porous silica material as an intermediate in the preparation of chemically bonded chromatographic stationary phases. Anal. Chem..

[101] Chu C.H., Jonsson E., Auvinen M., Pesek J.J., Sandoval J.E. (1993). A new approach for the preparation of a hydride-modified substrate used as an intermediate in the synthesis of surface-bonded materials. Anal. Chem..

[102] Pesek J.J., Matyska M.T., Oliva M., Evanchic M. (1998). Synthesis and characterization of bonded phases made via hydrosilation of alkynes on silica hydride surfaces. J. Chromatogr. A.

[103] Pesek J.J., Matyska M.T., Muley S. (2000). Synthesis and characterization of a new type of chemically bonded liquid crystal stationary phase for HPLC. Chromatographia.

[104] Kang K.K., Rhee H.K. (2005). Synthesis and characterization of novel mesoporous silica with large wormhole-like pores: Use of TBOS as silicon source. Microporous Mesoporous Mater..

[105] Rahmat N., Hamzah F., Sahiron N., Mazlan M., Zahari M.M. (2016). Sodium silicate as source of silica for synthesis of mesoporous SBA-15. IOP Conf. Ser. Mater. Sci. Eng..

[106] Wei F.Y., Liu Z.W., Lu J., Liu Z.T. (2010). Synthesis of mesoporous MCM-48 using fumed silica and mixed surfactants. Microporous Mesoporous Mater..

[107] Chen L., Xu J., Zhang W.H., Holmes J.D., Morris M.A. (2011). Syntheses of complex mesoporous silicas using mixtures of nonionic block copolymer surfactants: Understanding formation of different structures using solubility parameters. J. Colloid Interface Sci..

[108] Blin J.L., Otjacques C., Herrier G., Su B.L. (2000). Pore Size Engineering of Mesoporous Silicas Using Decane as Expander. Langmuir.

[109] Asefa T., MacLachlan M.J., Coombs N., Ozin G.A. (1999). Periodic mesoporous organosilicas with organic groups inside the channel walls. Nature.

[110] Inagaki S., Guan S., Fukushima Y., Ohsuna T., Terasaki O. (1999). Novel Mesoporous Materials with a Uniform Distribution of Organic Groups and Inorganic Oxide in Their Frameworks. J. Am. Chem. Soc..

[111] Melde B.J., Holland B.T., Blanford C.F., Stein A. (1999). Mesoporous Sieves with Unified Hybrid Inorganic/Organic Frameworks. Chem. Mater..

[112] Park S.S., Moorthy M.S., Ha C.S. (2014). Periodic mesoporous organosilicas for advanced applications. NPG Asia Mater..

[113] Goto Y., Mizoshita N., Waki M., Ikai M., Maegawa Y., Inagaki S. (2019). Synthesis and Applications of Periodic Mesoporous Organosilicas. Chemistry of Silica and Zeolite-Based Materials.

[114] Lintang H.O., Yuliati L. (2019). Designed Mesoporous Materials toward Multifunctional Organic Silica Nanocomposites. Mesoporous Materials—Properties and Applications.

[115] Huo Q., Margolese D.I., Stucky G.D. (1996). Surfactant Control of Phases in the Synthesis of Mesoporous Silica-Based Materials. Chem. Mater..

[116] Sullivan T.P., Huck W.T.S. (2003). Reactions on Monolayers: Organic Synthesis in Two Dimensions. Eur. J. Org. Chem..

[117] Haensch C., Hoeppener S., Schubert U.S. (2010). Chemical modification of self-assembled silane based monolayers by surface reactions. Chem. Soc. Rev..

[118] Lummerstorfer T., Hoffmann H. (2004). Click Chemistry on Surfaces: 1, 3-Dipolar Cycloaddition Reactions of Azide-Terminated Monolayers on Silica. J. Phys. Chem. B.

[119] Khung Y.L., Narducci D. (2015). Surface modification strategies on mesoporous silica nanoparticles for anti-biofouling zwitterionic film grafting. Adv. Colloid Interface Sci..

[120] Ziarani G.M., Hassanzadeh Z., Gholamzadeh P., Asadi S., Badiei A. (2016). Advances in click chemistry for silica-based material construction. RSC Adv..

[121] van den Berg S.A., Tu J., Sliedregt K.M., Kros A., Wennekes T., Zuilhof H. (2014). Clickable Mesoporous Silica via Functionalization with 1, omega-Alkenes. Adv. Mater. Interfaces.

[122] Lowe A.B. (2014). Thiol–ene “click” reactions and recent applications in polymer and materials synthesis: A first update. Polym. Chem..

[123] Wasserman S.R., Tao Y.T., Whitesides G.M. (1989). Structure and reactivity of alkylsiloxane monolayers formed by reaction of alkyltrichlorosilanes on silicon substrates. Langmuir.

[124] Ziarani G.M., Lashgari N., Badiei A. (2015). Sulfonic acid-functionalized mesoporous silica (SBA-Pr-SO3H) as solid acid catalyst in organic reactions. J. Mol. Catal. A Chem..

[125] Chaudhary V., Sharma S. (2017). An overview of ordered mesoporous material SBA-15: Synthesis, functionalization and application in oxidation reactions. J. Porous Mater..

[126] Laskowski L., Laskowska M. (2014). Functionalization of SBA-15 mesoporous silica by Cu-phosphonate units: Probing of synthesis route. J. Solid State Chem..

[127] Laskowski Ł., Laskowska M., Jelonkiewicz J., Galkowski T., Pawlik P., Piech H., Doskocz M. (2016). Iron doped SBA-15 mesoporous silica studied by Mössbauer spectroscopy. J. Nanomater..

[128] Laskowska M., Laskowski L., Jelonkiewicz J. (2015). SBA-15 mesoporous silica activated by metal ions–Verification of molecular structure on the basis of Raman spectroscopy supported by numerical simulations. J. Mol. Struct..

[129] Bałanda M., Pełka R., Fitta M., Laskowski Ł., Laskowska M. (2016). Relaxation and magnetocaloric effect in the Mn 12 molecular nanomagnet incorporated into mesoporous silica: A comparative study. RSC Adv..

[130] Laskowski L., Kityk I., Konieczny P., Pastukh O., Schabikowski M., Laskowska M. (2019). The Separation of the Mn12 Single-Molecule Magnets onto Spherical Silica Nanoparticles. Nanomaterials.

[131] Mannini M., Pineider F., Sainctavit P., Danieli C., Otero E., Sciancalepore C., Talarico A.M., Arrio M.A., Cornia A., Gatteschi D. (2009). Magnetic memory of a single-molecule quantum magnet wired to a gold surface. Nat. Mater..

[132] Park C.D., Jeong D.Y. (2001). Soluble Single-Molecule Magnet: Mn12-stearate. Bull. Korean Chem. Soc..

[133] Laskowska M., Pastukh O., Konieczny P., Dulski M., Zalsiński M., Laskowski L. (2020). Magnetic Behaviour of Mn12-Stearate Single-Molecule Magnets Immobilized on the Surface of 300 nm Spherical Silica Nanoparticles. Materials.

[134] Laskowska M., Oyama M., Kityk I., Marszalek M., Dulski M., Laskowski L. (2019). Surface functionalization by silver-containing molecules with controlled distribution of functionalities. Appl. Surf. Sci..

[135] Laskowska M., Pastukh O., Kuźma D., Laskowski Ł. (2019). How to Control the Distribution of Anchored, Mn12–Stearate, Single-Molecule Magnets. Nanomaterials.

[136] Pastukh O., Konieczny P., Czernia D., Laskowska M., Dulski M., Laskowski Ł. (2020). Aging effect on the magnetic properties of Mn12-stearate single-molecule magnets anchored onto the surface of spherical silica nanoparticles. Mater. Sci. Eng. B.

[137] Laskowski Ł., Laskowska M., Dulski M., Zubko M., Jelonkiewicz J., Perzanowski M., Vila N., Walcarius A. (2019). Multi-step functionalization procedure for fabrication of vertically aligned mesoporous silica thin films with metal-containing molecules localized at the pores bottom. Microporous Mesoporous Mater..

[138] Laskowski Ł., Laskowska M., Jelonkiewicz J., Boullanger A. (2015). Molecular Approach to Hopfield Neural Network. Proceedings of the International Conference on Artificial Intelligence and Soft Computing.

[139] Laskowski Ł., Laskowska M., Jelonkiewicz J., Piech H., Galkowski T., Boullanger A. (2016). The concept of molecular neurons. Proceedings of the International Conference on Artificial Intelligence and Soft Computing.

[140] Laskowska M., Laskowski Ł., Jelonkiewicz J., Piech H., Galkowski T., Boullanger A. (2017). Porous Silica Templated Nanomaterials for Artificial Intelligence and IT Technologies. Proceedings of the International Conference on Artificial Intelligence and Soft Computing.

[141] Dermont G., Bergeron M., Mercier G., Richer-Laflèche M. (2008). Soil washing for metal removal: A review of physical/chemical technologies and field applications. J. Hazard. Mater..

[142] Laskowski Ł., Laskowska M., Bałanda M., Fitta M., Kwiatkowska J., Dziliński K., Karczmarska A. (2014). Mesoporous silica SBA-15 functionalized by nickel–phosphonic units: Raman and magnetic analysis. Microporous Mesoporous Mater..

[143] Laskowska M., Kityk I., Dulski M., Jędryka J., Wojciechowski A., Jelonkiewicz J., Wojtyniak M., Laskowski Ł. (2017). Functionalized mesoporous silica thin films as a tunable nonlinear optical material. Nanoscale.

[144] Laskowski L., Laskowska M., Fijalkowski K., Piech H., Jelonkiewicz J., Jaskulak M., Gnatowski A., Dulski M. (2017). New class of antimicrobial agents: SBA-15 silica containing anchored copper ions. J. Nanomater..

[145] Laskowska M., Kityk I., Pastukh O., Dulski M., Zubko M., Jedryka J., Cpałka K., Zieliński P.M., Laskowski Ł. (2020). Nanocomposite for photonics—Nickel pyrophosphate nanocrystals synthesised in silica nanoreactors. Microporous Mesoporous Mater..

[146] Laskowski Ł., Majtyka-Piłat A., Cpałka K., Zubko M., Laskowska M. (2020). Synthesis in Silica Nanoreactor: Copper Pyrophosphate Quantum Dots and Silver Oxide Nanocrystallites Inside Silica Mezochannels. Materials.

[147] Laskowska M., Bałanda M., Fitta M., Dulski M., Zubko M., Pawlik P., Laskowski Ł. (2019). Magnetic behaviour of Mn12-stearate single-molecule magnets immobilized inside SBA-15 mesoporous silica matrix. J. Magn. Magn. Mater..

[148] Dulski M., Laskowska M., Sułowicz S., Krzykawski T., Pastukh O., Zieliński P., Pawlik P., Nowak A. (2020). The impact of the functionalization of silica mesopores on the structural and biological features of SBA-15. Microporous Mesoporous Mater..

[149] Laskowski L., Kassiba A., Makowska-Janusik M., Mehdi A., Gibaud A., Errien N., Swiatek J. (2009). Magnetic behaviour of nickel-cyclam complexes in mesoporous silica: EPR investigations. J. Phys. Condens. Matter.

[150] Laskowska M., Dulski M., Marszałek M., Zubko M., Laskowski Ł. (2019). Vertically aligned porous silica thin films functionalized by nickel chloride incorporated in walls. Microporous Mesoporous Mater..

