# Supplementary material for: Nanostructured Silica with Anchoring Units: The 2D Solid Solvent for Molecules and Metal Ions
Source: Int J Mol Sci. 2020 Oct 30;21(21):8137. doi: 10.3390/ijms21218137 (PMC7663599; doi:10.3390/ijms21218137)
Supplement: Supplementary file 1 [file ijms-21-08137-s001.pdf]

Int. J. Mol. Sci. **2020**, *21*, 1–7; doi:10.3390/ijms21218137

Supplementary Materials: Nanostructured Silica With Anchoring Units: The 2D Solid Solvent For Molecules And Metal Ions

Magdalena Laskowska, Oleksandr Pastukh, Andrii Fedorchuk, Mateusz Schabikowski, Paweł Kowalczyk, Marcin Zalasiński, and Łukasz Laskowski *

1. Procedures for the syntheses of silica-based nanostructured materials

All reagents were purchased at the highest possible purity.

1.1. *The synthesis of silica spheres with the diameter of 300 nm*

The procedure provided here is an optimized synthesis route applied by Stöber and his team in [1]. It is based on hydrolysis and polycondensation of tetraetyl orthosilicate (Sigma-Aldrich Chemie GmbH, Germany). The synthesis results in homogenous silica spheres with the diameter of 300 nm.

To obtain 0.6 g of spherical nanosilica the following reagents are required:

- deionized water: 10 cm^3;
- ethanol: 75 cm^3;
- TEOS: 5 cm^3;
- ammonia 25%: 4 cm^3.

The synthesis was performed in a 150 cm^3 polypropylene container with a cap. All procedures were carried out at ambient temperature. The process starts with mixing the solvents: water and ethanol under magnetic stirring. Next, TEOS was added and vigorously stirred for 10 min. After a clear solution was obtained, the ammonia was added as a catalyser of hydrolysis and polycondensation. The formation of spherical silica nanoparticles lasts for 10 hours under constant stirring. After the formation, the silica powder was recovered by centrifugation and washed a few times by ethanol and water taken from the excess of TEOS and ammonia. The powder was dried in vacuum at 120 °C for 24 h. The dry powder was stored in a tightly closed container in argon in order to avoid humidity (unwanted during functionalization).

1.2. *The functionalization of silica spheres with carboxyl and phosphoric acid groups*

The spherical silica can be functionalized by propyl carboxyl or propyl phosphoric acid units by a grafting procedure. This can be done with a full substitution of the surface hydroxyl units by functional groups or with controlled distribution of acidic molecules on the surface achieved by the application of spacers. All the procedures were described in our earlier works [2–4]. Here, we provide example procedures in detail.

Table S1. The amounts of precursors of anchoring units and spacer groups required to obtain the material with assumed number of spacer groups (N) per single anchor.

N	SilS-PO(OH)$_2$		SilS-COOH	
	PPTES [g]	TEOS [g]	BNTES [g]	TEOS [g]
0	3.573	0	2.313	0
1	1.786	1.042	1.157	1.042
6	0.500	1.792	0.324	1.792
12	0.286	1.917	0.185	1.917

The presented procedure is for complete functionalization (no spacer units, N=0) and the controlled distribution with 1, 6 or 12 spacer units separating anchoring groups (N=0, 1, 6 or 12, respectively). The samples are denoted as SilS-PO(OH)$_2$ NX and SilS-COOH NX (where X denotes number of spacer units per single anchoring groups) for samples containing phosphoric acid and carboxyl groups, respectively. This ratio was set by changing the proportions between the precursors of functional units (phosphonate propyl dietyl triethoxysilane – PPTES for phosphoric acid groups or butyronitrile triethoxysilane – BNTES – for carboxyl groups) and the precursor of spacer units, TEOS. The PPTES was purchased from Syntal Chemicals, Poland and BNTES from Sigma-Aldrich Chemie GmbH, Germany. The mass of reagents, shown in Table S1, was calculated from the molecular weight of the corresponding constituents (TEOS – 208.33 g/mol, PPTES – 357.29 g/mol, BNTES 231.36 g/mol).

The procedure consists of three steps. For the functionalization of 1.5 g of spherical silica, the following amounts of reagents are required:

Step 1:

- the spherical silica powder: 1.5 g;
- dichloromethane: 100 cm^3;
- TEOS: see: Table S1;
- PPTES / BNTES see: Table S1;.

Step 2:

- the pre-functionalized spherical silica powder: 1.5 g;
- dichloromethane: 50 cm^3;
- chlorotrimethyl silane (ClTMS, Sigma-Aldrich Chemie GmbH, Germany): 3 cm^3.

Step 3:

- pre-functionalized silylated spherical silica powder: 1.5 g;
- concentrated hydrochloric acid: 25 cm^3;
- acetone: 20 cm^3
- deionized water: 5 cm^3.

Prior to the synthesis, the silica powder was thoroughly dried in vacuum at 120 °C for at least 24 hours. In the first step, the precursors of anchoring groups and spacers were dissolved in dichloromethane and mixed for at least two hours (until a clear solution was obtained). This was done on a magnetic stirrer in a round flask under the protective atmosphere of argon. Next, dry spherical silica was added and the suspension was rigorously mixed under reflux for 24 hours. The pre-functionalized powder was recovered by centrifugation and washed several times with dichloromethane. After this procedure, the resulting powder was thoroughly dried in vacuum at the temperature of 120 °C for at least 24 hours and stored in a protective atmosphere.

In the next step (Step 2), the material was silylated in order to convert hydroxyl units into trimethyl silane groups (constituting spacers). For both types of materials, the procedure is the same. ClTMS was dissolved in dichloromethane, similarly to the previous step in, a round flask under a protective atmosphere. After obtaining clear solution (after approximately two hours), we added a dry pre-functionalized silica powder and stirred it under reflux for 24 h. The silylated powder was recovered by centrifugation and washed several times with dichloromethane. Next, the material was dried in vacuum at 120 °C for at least 24 hours.

In the last step, we hydrolyzed the precursors of anchoring units into acidic functional groups (phosphoric or carboxyl). For both types of materials, the procedure is the same. The silica powder was dispersed into a mixture of HCl, acetone and DI water. The addition of acetone is necessary because the

surface of silica spheres is strongly hydrophobic after silylation. The suspension was mixed under reflux for 24 h.The functionalized silica powder was recovered by centrifugation and washed several times by the mixture of DI water and acetone (till neutral pH was obtained). After drying in vacuum at 120 °C for at least 24 hours, the material is ready.

1.3. The synthesis of spherical mesoporous silica with MCM-41 architecture

In order to obtain spherical mesoporous silica with MCM-41 architecture, we optimized the procedure described in literature [5,6]. This route produces mesporous silica spheres with the diameter from 300 to 600 nm.

The procedure requires the following reagents:

- deionized water: 50 cm^3;
- ethanol: 50 cm^3;
- TEOS: 3.28 g;
- ammonia 25%: 14.56 cm^3.
- cetyl triethyl ammonium bromide – CTAB (Sigma-Aldrich Chemie GmbH, Germany)

In the first step, we dissolved CTAB in DI water and ethanol by mixing with a magnetic stirer. Next, we added TEOS to this mixture and stirred for 30 min until clear solution. Then, the ammonia was added to this solution as a catalyst, and we continued stirring for next three hours. The white precipitation occurred within this time. The solid product was recovered by centrifugation and washed several times by water. The CTAB template can be removed by washing with hot ethanol under soxhlet apparatus or by calcining the sample at 550 °C for 7h.

1.4. The synthesis of SBA-15 mesoporous silica containing phosphoric acid groups inside pores

The SBA-15 mesoporous silica containing phosphoric acid groups inside pores can be used for capturing two-valence metal ions. The specimen can be prepared via co-condensation with the assumed concentration of acidic groups inside pores in the presence of P123 non-ionic surfactant (poly(ethylene glycol)$_{20}$-poly(propylene glycol)$_{70}$-poly(ethylene glycol)$_{20}$ – Sigma-Aldrich Chemie GmbH, Germany). Here, we present the synthesis route on the example of four various concentrations of functional groups, defined by the N number – the number of mols of the precursor of silica (TEOS) per single mol of the precursor of functional units (PPTES in this case). The molar concentration of functional units refers to proportion between these units and silica molecules. To demonstrate, we provide the following N numbers: 9, 19, 39 and 79 which refer to the molar concentrations of: 10%, 5%, 2.5% and 1.25% of acidic groups.

The synthesis procedure is divided into three steps and requires the reagents listed below. The amounts of the precursors are provided in Table S2.

Step 1:

- deionised water pH=1.5 (HCl): 320 cm^3;
- P123 surfactant: 8 g;
- TEOS: see: Table S2;
- PPTES: see: Table S2;
- NaF (Sigma-Aldrich Chemie GmbH, Germany): 0.151 g

Step 2:

- pre-functionalized SBA-15 silica powder: 1.0 g;
- toluene: 50 cm^3;
- ClTMS: 1.2 g.

Table S2. The amounts of precursors of silica and anchoring units necessary for the fabrication of the functionalized SBA-15 silica with the assumed concentration of phosphoric acid groups defined by the N number

N ->	9		19		39		79	
precursor	mmols	grams	mmols	grams	mmols	grams	mmols	grams
TEOS	80.82	16.84	85.31	17.78	87.55	18.24	88.68	18.47
PPTES	8.98	3.08	4.49	1.54	2.25	0.77	1.12	0.38

Step 3:

- pre-functionalized silylated SBA-15 silica powder: 1.0 g;
- toluene: 50 cm^3;
- Bromotrimethyl silane (BrTMS, Sigma-Aldrich Chemie GmbH, Germany): 0.6 cm^3 (N=9), 0.4 cm^3 (N=19), 0.2 cm^3 (N=39), 0.1 cm^3 (N=79).
- methanol: 50 cm^3;

First, the solution of surfactant was prepared: P123 was dissolved in an aqueous solution of HCl (pH=1.5). After complete dissolution of the surfactant (after approximately five hours), we added weighted amounts of the precursors, TEOS and PPTES, during vigorous magnetic stirring. It is important to maintain high-speed stirring during this process in order to avoid the polymerization of the precursors. This mixture was stirred at room temperature till the clear solution was obtained (after approximately three hours). Next, a small amount of NaF was added in order to induce hydrolysis and polycondensation and the material was immediately heated to 60 °C in hot oil bath and stirred vigorously for 48 hours. The resulting material was filtered and placed in a Soxhlet apparatus in order to remove the surfactant by hot ethanol. The obtained powder was dried in vacuum at 120 °C for 24 h.

In the second step (Step 2), the silylation was done to avoid unwanted side reactions. It was realized by treating the dry pre-functionalyzed mesoporous powder with ClTMS in toluene under reflux for 24 h. After this process, the material was filtered and washed several times with toluene and acetone. Next, the silica powder was dried similarly like in the previous step.

For the hydrolysis of ester phosphoric acid groups into phosphoric acid groups, we applied a two-step selective hydrolysis procedure (Step 2). To do this, we treated the dry silylised pre-functionalyzed mesoporous powder with BrTMS in toluene under reflux for 24 h. Afterwards, the material was filtered and washed several times with toluene and acetone. In order to complete the hydrolysis, the powder was mixed with methanol and placed under reflux for 5 h. Next, the silica powder was filtered and dried.

1.5. The synthesis of SBA-15 mesoporous silica containing carboxyl groups inside pores

The synthesis of the SBA-15 mesoporous silica containing carboxyl groups inside pores is a similar technique to the one described above. As in the previous description, we provide the following N numbers: 9, 19, 39 and 79 referring to the molar concentrations of: 10%, 5%, 2.5% and 1.25% of acidic groups. The first two steps (Step 1 and Step 2) are analogous to the procedure for the material containing phosphoric acid groups with the exception of a different precursor of functional unit: BNTES (instead of PPTES).

The synthesis is divided into three steps and requires the reagents listed below. The amounts of the precursors are listed in Table S3.

Step 1:

- deionised water with pH=1.5 (HCl): 320 cm^3;
- P123 surfactant: 8 g;
- TEOS: see Table S3;

Table S3. The amounts of precursors of silica and anchoring units necessary for the fabrication of the functionalized SBA-15 silica with the assumed concentration of carboxyl groups defined by the N number

N ->	9		19		39		79	
precursor	mmols	grams	mmols	grams	mmols	grams	mmols	grams
TEOS	80.82	16.84	85.31	17.78	87.55	18.24	88.68	18.47
BNTES	8.98	2.08	4.49	1.04	2.25	0.52	1.12	0.26

- BNTES: see Table S3;
- NaF: 0.151 g

Step 2:

- pre-functionalized SBA-15 silica powder: 1.0 g;
- toluene: 50 cm^3;
- ClTMS: 1.2 g.

Step 3:

- pre-functionalized silylated spherical silica powder: 1.0 g;
- concentrated hydrochloric acid: 40 cm^3;
- acetone: 20 cm^3
- deionized water: 10cm^3.

Steps 1 and 2 are the same as described for the previous material, thus we direct the readers to the section 1.4.

In the last step (STEP 3), we hydrolysed the cyanopropyl units into carboxyl groups. The silica powder was dispersed in a mixture of HCl, acetone and DI water. The addition of acetone was necessary to overcome the hydrophobity of the surface of SBA-15 silica. The suspension was mixed under reflux for 24 h. The functionalized mesoporous silica powder was filtered and washed several times by the mixture of DI water and acetone (1:1) till neutral pH was obtained. After drying in vacuum at 120 °C for at least 24 hours, the material was ready.

1.6. The synthesis of SBA-15 mesoporous silica containing cyclam molecules inside pores

Also in this case, we applied the co-condensation method to obtain SBA-15 silica with cyclam molecules (1,4,8,11-Tetraazacyclotetradecane) attached inside pores via propyl chain. Similarly as before, to define correctly the doping rates, we refer to the molar ratios between chloropropyl trimethoxysilane (ClPTMOS, Sigma-Aldrich Chemie GmbH, Germany) as a precursor for cyclam and the silica units from TEOS. We provide examples for the molar concentrations of: 10%, 5%, 2.5% and 1.25% of cyclam-containing groups (N = 9, 19, 39, and 79).

The procedure is divided into two steps requiring following reagents:

Step 1:

- deionised water with pH=1.5 (HCl): 320 cm^3;
- P123 surfactant: 8 g;
- TEOS: see Table S4;
- ClPTMOS: see Table S4;
- NaF: 0.151 g

Step 2:

- pre-functionalized SBA-15 silica powder: 1.0 g;

Table S4. The amounts of precursors of silica and anchoring units necessary for the fabrication of the functionalized SBA-15 silica with the assumed concentration of chloropropyl groups defined by the N number

N ->	9		19		39		79	
precursor	mmols	grams	mmols	grams	mmols	grams	mmols	grams
TEOS	80.82	16.84	85.31	17.78	87.55	18.24	88.68	18.47
ClPTMOS	8.98	1.78	4.49	0.89	2.25	0.47	1.12	0.23

Table S5. The amounts of precursors of silica and anchoring units necessary for the fabrication of the functionalized SBA-15 silica with the assumed concentration of chloropropyl groups defined by the N number

N ->	6		16		36		76	
precursor	mmols	grams	mmols	grams	mmols	grams	mmols	grams
TEOS	76.97	16.04	84.52	17.60	87.37	18.20	88.63	18.46
TS-cyclam	12.83	13.04	5.28	5.36	2.43	2.47	1.17	1.19

- cyclam (Sigma-Aldrich Chemie GmbH, Germany): 0.65 g (N=9), 0.35 g (N=19), 0.18 g (N=39), 0.09 g (N=79).
- acetonitrile: 45 cm^3.
- triethylamine: 1 cm^3.

The first step of the synthesis (Step 1) depends on the polycondensation of TEOS and ClPTMOS in the presence of P123 surfactant and results in SBA-15 silica containing chloropropyl units inside pores. The procedure is the same as the one described in section 1.4.

In the second step (Step 2), the chlorine atoms in chlopropyl gropus are substituted by cyclam. This is realized by mixing the pre-functionalized silica powder with cyclam dissolved in acetonitrile and trietylamine. The product was heated under reflux and stirred for 2 days. The white solid can be recovered by filtration and washing five times with hot chloroform and three times with hot ethanol to remove the excess of cyclam. After drying at 120 °C overnight in vacuum, the material is ready.

1.7. The synthesis of SBA-15 mesoporous silica containing cyclam molecules in structure

The SBA-15 mesoporous silica containing cyclam molecules incorporated in silica walls can be obtained in a single-step reaction of polycondensation of TEOS and 1,4,8,11 trietoxysilane propyl 1,4,8,11-tetraazacyclotetradecane (tetrasilylated cyclam, hereafter called TS-cyclam). In this case, however, the molar proportions between cyclam and silica units must be calculated differently since precursor of cyclam contains four atoms of silicon. Here, the proportions of 1 mol of TS-cyclam per 6 mols of TEOS involves 10% cyclam of molar. Analogically: 1:16, 1:36 and 1:76 correspond to the molar concentrations of cyclam of: 5%, 2.5% and 1.25%

The synthesis procedure involves the reagents listed below. The amounts of the precursors are shown in Table S5.

- deionised water, pH=1.5 (adjusted with HCl): 320 cm^3;
- P123 surfactant: 8 g;
- TEOS: see Table S5;
- TS-cyclam (Syntal Chemicals, Poland,): see Table S5;
- NaF (Sigma-Aldrich Chemie GmbH, Germany): 0.151 g

The preparation procedure depends on polycondensation of TEOS and TS-cyclam in the presence of P123 surfactant. The synthesis route is the same as described in section 1.4. After the removal of the surfactant by hot ethanol under Soxhlet apparatus and drying, the material is ready

Abbreviations

The following abbreviations were used in this manuscript:

TEOS	tetraethyl ortosilicate
PPTES	phosphonate propyl dietyl triethoxysilane
BNTES	butyronitrile triethoxysilane / cyanopropyl triethoxysilane
ClTMS	chlorotrimethyl silane
ClPTMOS	chloropropyl trimethoxysilane
cyclam	1,4,8,11-Tetraazacyclotetradecane
P123	(poly(ethylene glycol)$_{20}$-poly(propylene glycol)$_{70}$-poly(ethylene glycol)$_{20}$
CTAB	cetyl triethyl ammonium bromide
TS-cyclam	tetrasilylated cyclam, 1,4,8,11 trietoxysilane propyl 1,4,8,11-tetraazacyclotetradecane

References

1. Stöber, W.; Fink, A.; Bohn, E. Controlled growth of monodisperse silica spheres in the micron size range. *Journal of colloid and interface science* **1968**, *26*, 62–69.
2. Laskowska, M.; Oyama, M.; Kityk, I.; Marszalek, M.; Dulski, M.; Laskowski, L. Surface functionalization by silver-containing molecules with controlled distribution of functionalities. *Applied Surface Science* **2019**, *481*, 433–436.
3. Laskowski, L.; Kityk, I.; Konieczny, P.; Pastukh, O.; Schabikowski, M.; Laskowska, M. The Separation of the Mn12 Single-Molecule Magnets onto Spherical Silica Nanoparticles. *Nanomaterials* **2019**, *9*, 764.
4. Laskowska, M.; Pastukh, O.; Kuźma, D.; Laskowski, Ł. How to Control the Distribution of Anchored, Mn12–Stearate, Single-Molecule Magnets. *Nanomaterials* **2019**, *9*, 1730.
5. Meléndez-Ortiz, H.; García-Cerda, L.; Olivares-Maldonado, Y.; Castruita, G.; Mercado-Silva, J.; Perera-Mercado, Y. Preparation of spherical MCM-41 molecular sieve at room temperature: Influence of the synthesis conditions in the structural properties. *Ceramics International* **2012**, *38*, 6353–6358.
6. Etienne, M.; Lebeau, B.; Walcarius, A. Organically-modified mesoporous silica spheres with MCM-41 architecture. *New Journal of Chemistry* **2002**, *26*, 384–386.

Publisher's Note: MDPI stays neutral with regard to jurisdictional claims in published maps and institutional affiliations.
